# The pre-Pleistocene fossil thylacinids (Dasyuromorphia: Thylacinidae) and the evolutionary context of the modern thylacine

**DOI:** 10.7717/peerj.7457

**Published:** 2019-09-02

**Authors:** Douglass S. Rovinsky, Alistair R. Evans, Justin W. Adams

**Affiliations:** 1 Department of Anatomy and Developmental Biology, Monash University, Clayton, VIC, Australia; 2 School of Biological Sciences, Monash University, Clayton, VIC, Australia; 3 Geosciences, Museums Victoria, Melbourne, VIC, Australia

**Keywords:** Tasmanian tiger, *Thylacinus cynocephalus*, Marsupial, Hypercarnivory, Body mass, Parsimony, Phylogeny, Middle Miocene climatic transition

## Abstract

The thylacine is popularly used as a classic example of convergent evolution between placental and marsupial mammals. Despite having a fossil history spanning over 20 million years and known since the 1960s, the thylacine is often presented in both scientific literature and popular culture as an evolutionary singleton unique in its morphological and ecological adaptations within the Australian ecosystem. Here, we synthesise and critically evaluate the current state of published knowledge regarding the known fossil record of Thylacinidae prior to the appearance of the modern species. We also present phylogenetic analyses and body mass estimates of the thylacinids to reveal trends in the evolution of hypercarnivory and ecological shifts within the family. We find support that *Mutpuracinus archibaldi* occupies an uncertain position outside of Thylacinidae, and consider *Nimbacinus richi* to likely be synonymous with *N. dicksoni*. The Thylacinidae were small-bodied (< ~8 kg) unspecialised faunivores until after the ~15–14 Ma middle Miocene climatic transition (MMCT). After the MMCT they dramatically increase in size and develop adaptations to a hypercarnivorous diet, potentially in response to the aridification of the Australian environment and the concomitant radiation of dasyurids. This fossil history of the thylacinids provides a foundation for understanding the ecology of the modern thylacine. It provides a framework for future studies of the evolution of hypercarnivory, cursoriality, morphological and ecological disparity, and convergence within mammalian carnivores.

## Introduction

The thylacine (*Thylacinus cynocephalus* Harris, 1808) is arguably a modern Australian icon. Since the last known individual died in captivity in 1936, it has served as a cautionary tale of the impact of humans on ecosystems and a potent symbol for the conservation of endangered species (Moeller, 1997; Paddle, 2000; Wemmer, 2002; Prowse et al., 2013; Barnett & Lorenzen, 2018). Recent interest in the thylacine has produced a slew of media activity surrounding the animal, including (in brief) popular books (Freeman, 2014), popular-science conference presentations (Windle, 2017), and even a full-length motion picture (Nettheim, 2011). This is in addition to peer-reviewed scholarly output, which has also seen a recent increase in number of publications and of researcher interest (Sleightholme & Campbell, 2016; Berns & Ashwell, 2017; Feigin et al., 2017; Haygarth, 2017; Newton, Feigin & Pask, 2017; White, Mitchell & Austin, 2017; White et al., 2018).

While these studies have brought about intriguing results, they are hampered by the fact that the thylacine was the only member of the family Thylacinidae to persist until modern times. In effect this presents the thylacine as a one-off, singular organism unique in its evolution and traits, a concept highlighted in many studies (Miller et al., 2009; Menzies et al., 2012; Feigin et al., 2017; Newton et al., 2018). Although this seeming uniqueness has prompted much research into the thylacine, of which the examples given here are only a small portion, it can potentially hinder efforts or present false signals when attempting to understand its ecology or behaviour.

The thylacine last shared a common ancestor with extant dasyuromorphians ~38.2 Ma (Dos Reis et al., 2012; Mitchell et al., 2014; Westerman et al., 2016; Kealy & Beck, 2017). This deep split in evolutionary history between thylacines and their closest extant relatives/marsupial analogues is similar in depth to, e.g., that separating the drastically disparate meerkat (*Suricata suricatta*) and tiger (*Panthera tigris*) – ~37.1 Ma (Eizirik et al., 2010; Zhou, Wang & Ma, 2017). This separation makes it difficult to draw tightly constrained conclusions regarding the ecologic niche of the thylacine. Additionally, their superficial similarity to canids and difference from any living marsupial has frustrated analyses and often led to dissimilar results. As an example, recent studies have found remarkable levels of morphologic similarity and convergence between thylacines and placental analogues (Feigin et al., 2017; Newton, Feigin & Pask, 2017). Conversely, other research has served to underscore how distinct they were from perceived analogues, both functionally and presumably ecologically (Jones & Stoddart, 1998; Jones, 2003; Wroe et al., 2007; Attard et al., 2011; Figueirido & Janis, 2011; Janis & Figueirido, 2014).

**Figure 1:**
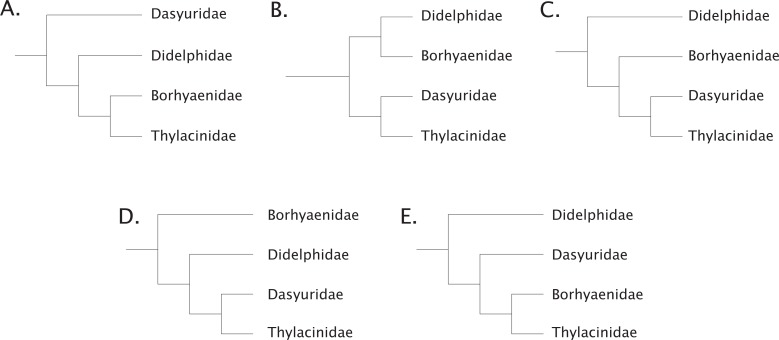
Historical concepts of the interrelationships between the carnivorous metatherians. (A) after Sinclair (1905), Wood (1924); (B) after Matthew (1915), Pocock (1926), Simpson (1941); (C) after Cabrera (1927); (D) after Marshall (1977); (E) after Marshall (1977), Archer (1982).

To add to these difficulties, recent phylogenetic work has radically refined our understanding of the time-depth of the dasyuromorphian radiations along with the position of the thylacinids within the clade (Meredith et al., 2009; May-Collado, Kilpatrick & Agnarsson, 2015; Archer et al., 2016; Westerman et al., 2016; Kealy & Beck, 2017). The understanding of the relationship of the thylacinids to the other Australian marsupials has had a convoluted history. Sinclair (1905) considered the thylacine to be a derived member of what was then known as the borhyaenids, which are now found to be members of various lineages within the South American non-marsupial metatherian Order Sparassodonta (Forasiepi, 2009; Ladeveze & De Muizon, 2010; Beck et al., 2014; Forasiepi, Judith Babot & Zimicz, 2015; Suarez et al., 2016; Wilson et al., 2016). These taxa (e.g., *Borhyaena, Prothylacinus, Cladosictis*, and *Sipalocyon* (*Amphiproviverra*)) were grouped by Sinclair (1905) with the thylacinids on the basis of various dental and postcranial characters. Wood (1924) supported this view, but various dissenting hypotheses were prevalent (Matthew, 1915; Pocock, 1926; Cabrera, 1927; Fig. 1). Both Matthew (1915) and Simpson (1941) argued that the dental characters linking the two groups were most likely due to a remarkable degree of convergence on hypercarnivory, although authors as late as Archer (1976) posited the possibility of a common ancestor for the two to the exclusion of the dasyurids. Marshall (1977) suggested that the apparent affinities of the thylacinids and borhyaenids were due to strong convergence, and argued for the thylacinids to be allied with the dasyurids. It was not until the early 1980s that molecular evidence would lend support to an increasing number of morphologic studies confirming the inclusion of Thylacinidae within Dasyuromorphia (Marshall, 1977; Archer, 1982; Sarich, Lowenstein & Richardson, 1982; Szalay, 1982). Within Dasyuromorphia the position of Thylacinidae has been recovered as either the sister-group to the dasyurids with a deeper Myrmecobiidae (Krajewski, Wroe & Westerman, 2000; Wroe & Musser, 2001) or as sister to both Dasyuridae and Myrmecobiidae (Miller et al., 2009). Recently, consensus has coalesced around the latter hypothesis, with Thylacinidae sister to a clade formed by Myrmecobiidae + Dasyuridae (May-Collado, Kilpatrick & Agnarsson, 2015; Westerman et al., 2016; Kealy & Beck, 2017).

Our understanding of the thylacinid phylogeny has been greatly aided by dramatic shifts in our knowledge of fossil thylacinids (Wroe, 2003; Yates, 2015). The first pre-Pleistocene fossil thylacinid (i.e., not *Thylacinus cynocephalus*) was not described until the end of the 1960s (Woodburne, 1967). It would be over two decades until a second fossil thylacinid was described (Muirhead & Archer, 1990). From that point no fewer than ten additional taxa have been described, with new species described as recently as 2015 (Yates, 2015). This had greatly increased our ability to place the recently extinct thylacine into an evolutionary context, and has underscored the deep divergence and potential evolutionary distance between the thylacine and its closest extant relatives.

We aim to provide a synthesised review of the current state of knowledge of the pre-Pleistocene fossil Thylacinidae, including biological and ecological implications of their evolutionary history and gaps in our knowledge. It is hoped that by highlighting the history of the thylacine, future studies may benefit from contextualising their research within this framework. This evolutionary history underscores that the thylacine was not a unique organism, but like all organisms part of an evolutionary radiation with clear trends towards what would ultimately be represented in *Thylacinus cynocephalus*.

## Materials and Methods

**Figure 2:**
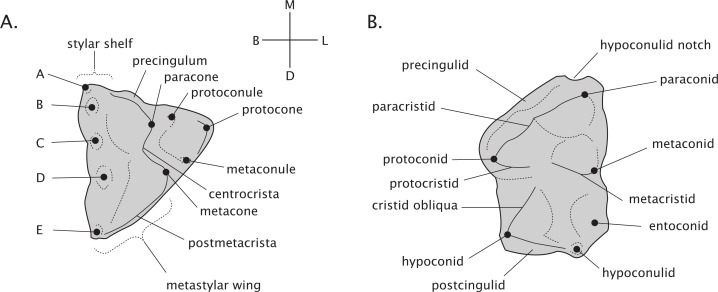
Dental terminology of generalised marsupial molars. (A) Right maxillary molar. (B) Left mandibular molar. Labels “A–E” refer to stylar cusps.

Adult thylacinid dental formula is 4.1.3.4/3.1.3.4 following the premolar/molar boundary of Flower (1867) and Luckett (1993). Terminology of molar morphology is presented in Fig. 2, largely following Kielan-Jaworowska, Cifelli & Luo (2004; pp. 412, 434). Our use of the terms faunivory and hypercarnivory refer to specific derivations within general carnivore ecology. Faunivory here refers to dental morphology suggesting a subequal consumption of invertebrate and vertebrate prey, without any specialised trends towards either insectivory or the heavy consumption of vertebrate flesh. Hypercarnivory is generally defined as a diet consisting of >70% vertebrate flesh (Van Valkenburgh, 2007), but as the dietary habits of fossil taxa are unobservable, is here used to refer to any dental trend towards the simplification of the carnassial and molar teeth and lengthening of their cutting blades, effectively increasing their shearing and reducing their grinding capabilities.

The phylogenetic history of Thylacinidae was explored via both parsimony and Bayesian Mkv models. We compiled a morphological dataset modified from that of Yates (2014) and Kealy & Beck (2017) comprising 80 characters scored across 25 taxa, including all currently described thylacinids. Where characters for fossil thylacinids needed scoring, or to cross-check previous scores, published descriptions and specimen photographs were used, as the physical specimens were unavailable for examination. The parsimony analysis was performed in tree analysis using new technology (TNT) v. 1.5 (Goloboff, Farris & Nixon, 2008). A skeleton constraint tree for the extant dasyurids was constructed in Mesquite v. 3.51 based on recent molecular phylogenies (Archer et al., 2016; Westerman et al., 2016; Kealy & Beck, 2017; Maddison & Maddison, 2018). As the number of taxa was relatively low, the analysis was conducted via implicit enumeration. Support for the tree nodes was assessed in TNT by calculating the Bremer support on suboptimal trees, and group present/contradicted frequency values generated by symmetric resampling (*P* = 33) at 5,000 replicates, using tree bisection reconnection. The resultant consensus tree was time-scaled with the Bell & Lloyd (2014) R package ‘strap’ using the ‘equal’ methodology adapted therein from Brusatte et al. (2008). Age ranges for the fossil taxa are based on published literature and references for the non-thylacinid taxa given in Dataset S4, with justifications for the thylacinids provided below in Systematic Palaeontology.

Regarding thylacinid intrafamilial relationships, the middle Miocene taxon *Mutpuracinus archibaldi* was initially found to be a plesiomorphic member of Thylacinidae (Murray & Megirian, 2000, 2006a; Wroe, 2003; Yates, 2014, 2015). However, recent work has grouped the taxon with the plesiomorphic dasyuromorphian *Barinya wangala*, both of which have been shown to consistently fall outside Thylacinidae (Archer et al., 2016; Kealy & Beck, 2017; Travouillon & Phillips, 2018). To assess the hypothesis that *Mutpuracinus archibaldi* is a thylacinid, an altered skeleton tree was used along with the ‘*force*’ command to produce this constraint, and a second parsimony analysis was run. A Templeton test was performed in TNT against both the resultant consensus trees as well as against the first most parsimonious tree for each analysis to determine the significance of any difference.

The Bayesian analyses were performed in MrBayes v. 3.2.6 on the CIPRES Science Gateway (Miller, Pfeiffer & Schwartz, 2010; Ronquist et al., 2012). An undated Mkv + G model analysis was performed on the morphological matrix, with a root, ingroup, and Phascogalini constraint topology to serve as an analogue to the skeleton tree used in the TNT analysis above. This analysis was comprised of four runs of four chains, sampling every 5,000 generations, for 20 million generations, generating a post-burn-in 50% majority rules consensus tree.

A tip-dating analysis was performed on the morphological matrix with the addition of ribosomal 12S RNA molecular data. The single gene was chosen as we had complete coverage of our extant taxa, and as to limit the potential of overwhelming the small set of morphological data, especially considering that our study clade is near-entirely devoid of genomic data. The molecular data and partitioning scheme was taken from Kealy & Beck (2017), and Genbank accession numbers provided in Table S3. A single independent gamma rates clock model was used and a fossilised birth–death prior assumed. Extant sampling was set to ‘random’ as we had an unequal sampling of extant dasyurids, with a sample probability of 0.0833 for the extant taxa. Default speciation, extinction, and fossilisation priors were used, and extant terminal taxa were assigned an age of zero Ma. Age range justification was the same as that used in the parsimony tree time-scaling above. The resulting ‘allcompat’ consensus tree was then also time-scaled with the R package ‘strap’ as a separate methodological comparison.

Body mass estimates for all published pre-Pleistocene thylacinid specimens known from craniodental material were calculated from the dasyuromorphian-only regression equations developed by Myers (2001). Body mass estimates for several of the pre-Pleistocene thylacinids have been published (Wroe, 2001; Travouillon et al., 2009; Yates, 2014, 2015), but a comprehensive set of estimates using all applicable published specimens has yet to be performed. The highest ranked variable possible was chosen for each specimen, and as each specimen was therefore potentially subject to a different calculation, individual values are given in lieu of mean values for species. Regarding the large *Thylacinus megiriani* and *Thylacinus potens*, dental metrics exceed that covered by the equations of Myers (2001). Wroe (2001) discussed this and noted that both taxa appear to vary in their dental proportions from the extant species, and chose to use geometric similitude with *Thylacinus cynocephalus* to estimate the mass of these large thylacinids. We here instead choose to employ the equations of Myers (2001), firstly as there is no a priori reason to view geometric similitude as necessarily a more accurate estimate, given the noted proportional difference between the taxa, and secondly to give a comparative set of estimates to be complementary to those of Wroe (2001).

All data matrices, R code, and body mass estimates are presented in the Supplemental Material.

The dating of Australian fossil localities has been a slow and difficult process, with the majority of sites having no associated absolute ages. Dating the Australian fossil sites has historically been based on biochronology, with many papers seeking to stratify, order, and correlate the localities (Archer et al., 1989; Murray & Megirian, 1992; Travouillon et al., 2006; Megirian et al., 2010; Arena et al., 2015). Recent work has begun to provide radiometric dates for some sites (Woodhead et al., 2016). We have attempted to correlate sites and specimens with dates wherever possible, using biocorrelation seriations correlated with sites that now have been quantitatively dated. However, the quantitative dating of Australian fossil localities is very much in its infancy, so we stress that the site dates and taxa age ranges given here are estimates.

## Results

### Phylogenetic analysis

**Figure 3:**
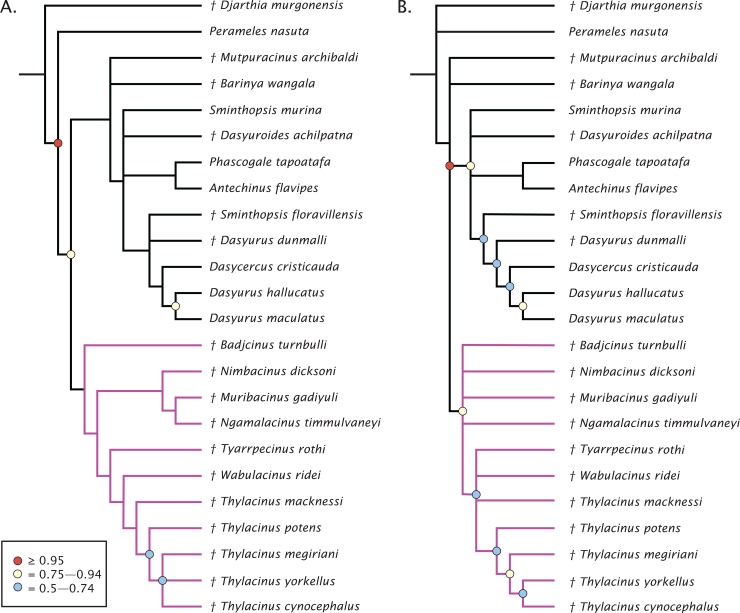
Phylogenetic results based on the 80 character morphological dataset. (A) Majority rules 50% consensus of 30 most parsimonious trees under implicit enumeration (length 171, CI: 0.520, RI: 0.645) after removal of *Maximucinus muirheadae* from the matrix. (B) Bayesian analysis majority rules 50% consensus of post-burn-in trees. Branch lengths are arbitrary. Circles at nodes are bootstrap GC values in (A), BPP in (B), only values ≥50% are shown. Thylacinidae in magenta.

The undated parsimony and Bayesian phylogenetic results are presented in Fig. 3. The parsimony analysis using implicit enumeration recovered 30 most parsimonious trees (length 171, CI: 0.520, RI: 0.645), after a posteriori removal of *Maximucinus muirheadae* (85% missing characters; Fig. 3A). Bremer support values higher than 1.0 were found for all nodes, though bootstrap values equal to or higher than 50% were only recovered for *Perameles* + Dasyuromorphia (100), extant *Dasyurus* (excluding *Dasyurus dunmalli*; 78), a clade comprising *Thylacinus* spp. excluding *Thylacinus macknessi* (51), and a polytomy containing *Thylacinus megiriani*, *Thylacinus yorkellus*, and *Thylacinus cynocephalus* (60). The forcing of *Mutpuracinus archibaldi* into Thylacinidae resulted in a slightly longer tree (length = 174, CI: 0.511, RI: 0.6432), indicating less support for that hypothesis, although both Templeton tests found the difference to be not significant.

Bayesian analysis of the morphological data recovers a very similar, though less resolved topology (Fig. 3B). Monophyly of Dasyuridae received higher support here (Bayesian posterior probability (BPP) = 0.83). As with the parsimony analysis, placement of the fossil dasyurids did not support their generic attributions, though with the necessary caveat that the current study was not designed to uncover the relationships within Dasyuridae. Thylacinidae receives moderate supports (BPP = 0.84), though the intrafamilial relationships are highly unresolved and only weakly supported, comprised of a basal polytomy, a polytomy containing *Tyarrpecinus, Wabulacinus*, and *Thylacinus macknessi* (BPP = 0.67), and a clade containing the remainder of *Thylacinus* spp. (BPP = 0.66). The only thylacinid clade here with a relatively strong support is that containing *Thylacinus megiriani*, *Thylacinus yorkellus*, and *Thylacinus cynocephalus* (BPP = 0.91).

**Figure 4:**
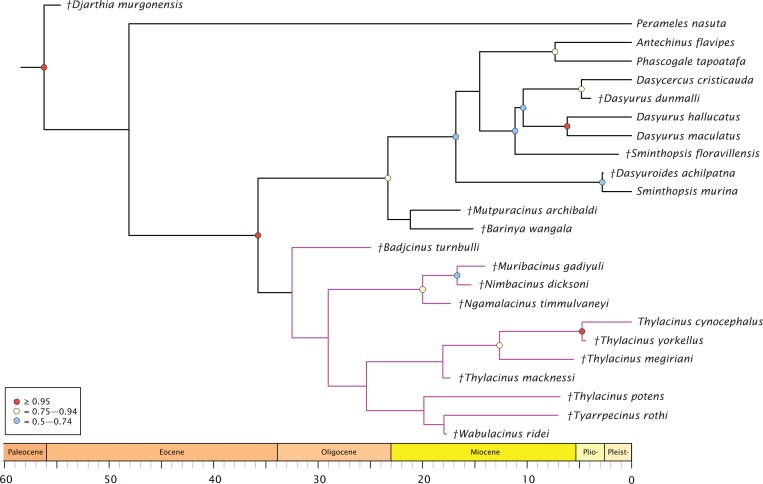
Phylogenetic tip-dated results based on the 80 character morphological and 853 bp 12S RNA sequence data. Majority rule consensus of post-burn-in trees showing all compatible clades (‘allcompat’ function in MrBayes) after removal of *Maximucinus muirheadae* from the matrix. Circles at nodes are BPP, only values ≥50% are shown. Thylacinidae in magenta.

The molecular + morphology tip-dated Bayesian analysis is presented in Fig. 4. Again, a monophyletic Dasyuridae is supported, but less strongly (BPP = 0.73). A clade consisting of *Mutpuracinus, Barinya*, and Dasyuridae is relatively well supported (BPP = 0.84) although the placement of these two taxa within this clade is uncertain, with little support for their sister relationship (BPP = 0.34). Within Dasyuridae, *Sminthopsis floravillensis* and *Dasyurius dunmalli* again cluster with Dasyurini, although with minimal support (BPP = 0.55). A surprising result is the clade comprised of *Sminthopsis murina* and *Dasyuroides achilpatna* (BPP = 0.73), a topology not recovered by Kealy & Beck (2017) or Archer et al. (2016). Thylacinidae here is unsupported (BPP = 0.29), with only three internal nodes receiving even marginal support: a clade containing *Ngamalacinus, Nimbacinus*, and *Muribacinus* (BPP = 0.80), a clade containing *Thylacinus megiriani, Thylacinus yorkellus*, and *Thylacinus cynocephalus* (BPP = 0.80), and the sister relationship of *Thylacinus yorkellus* and *Thylacinus cynocephalus* (BPP = 0.95). A very interesting result is the non-monophyly of *Thylacinus*, caused by the exclusion of *Thylacinus potens*. This result is not recovered in the other analyses and is not well supported here.

The split of Thylacinidae from the rest of Dasyuromorphia was estimated to be 41.8–35.7 Ma, comparable to that of past studies (range of ~43.0–30.7 Ma; Dos Reis et al., 2012; Mitchell et al., 2014; Westerman et al., 2016; Kealy & Beck, 2017; see Fig. S1). The internal structure of Thylacinidae is poorly resolved, but some timing can be hypothesised. *Badjcinus* appears to have split from the rest of Thylacinidae approximately 34.8–32.5 Ma, with the *Ngamalacinus + Nimbacinus + Muribacinus* clade again splitting 29.0–25.9 Ma. The clade including *Tyarrpecinus + Thylacinus cynocephalus* originated 24.1–25.4 Ma, and the clade including *Thylacinus megiriani*, *Thylacinus yorkellus*, and *Thylacinus cynocephalus* approximately 13.5–8.0 Ma.

The analyses all support placing *Mutpuracinus archibaldi* outside of Thylacinidae as potentially sister to Dasyuridae or as a stem dasyuromorph, similar to placements recovered by Kealy & Beck (2017) and Travouillon & Phillips (2018). *Badjcinus turnbulli* is generally recovered as sister to the remainder of Thylacinidae, with a topology further separated into two internal clades—one comprised of plesiomorphic, small-bodied thylacinids (*Nimbacinus, Muribacinus, Ngamalacinus*) and one consisting of *Tyarrpecinus, Wabulacinus*, and *Thylacinus*. This is similar to topologies recovered in previous work (Yates, 2015; Archer et al., 2016; Kealy & Beck, 2017).

### Body mass

Body mass estimates for the 35 applicable specimens are given in Table S2. *Badjcinus turnbulli* is estimated to be between 1.7 and 3.1 kg, roughly comparable to the modern *Dasyurus maculatus*. Both specimens of *Muribacinus gadiyuli* are small, at 1.6 and 1.7 kg, respectively, while *Maximucinus muirheadae* is surprisingly large (18.4 kg) as stated in Wroe (2001). Both *Ngamalacinus timmulvaneyi* and *Nimbacinus dicksoni* are slightly larger than extant quolls, at 5.7–8.4 and 2.9–6.8 kg, respectively. *Tyarrpecinus rothi* and *Wabulacinus ridei* are also estimated to be of similar size at 5.4 and 5.3–7.8 kg, respectively.

The members of the genus *Thylacinus* are larger as a whole than the other taxa within the family. The small and early-diverging *Thylacinus macknessi* is estimated at 6.7–9.0 kg. The large-bodied thylacinids *Thylacinus megiriani* and *Thylacinus potens* are estimated to be 49.1 and 28.3–55.0 kg, respectively, and *Thylacinus yorkellus* at 14.5–17.8 kg. Recorded masses for the recent *Thylacinus cynocephalus* are scant, but the estimated range of 15–35 kg by Moeller (1970) is likely accurate.

## Systematic palaeontology

**Class** Mammalia Linnaeus, 1758
**Subclass** Theria Parker & Haswell, 1897
**Infraclass** Metatheria Huxley, 1880
**Supercohort** Marsupialia (Illiger, 1811) Cuvier, 1817
**Cohort** Australidelphia Szalay, 1982
**Order** Dasyuromorphia Gill, 1872
**Dasyuromorphia *incertae sedis***
**Genus** *Mutpuracinus* Murray & Megirian, 2000
***Mutpuracinus archibaldi*** Murray & Megirian, 2000

**Holotype:** NTM P907-3, partial left maxilla.

**Type Locality:** Bullock Creek Local Fauna (LF), Blast Site, Northern Territory, Australia.

**Referred Specimens:** see Table S1

**Distribution and Age:** The Bullock Creek LF in Northern Territory is currently undated, but has been estimated to be Camfieldian (~17–12 Ma) sensu Megirian et al. (2010).

**Diagnosis:** Following the amended diagnosis by Murray & Megirian (2006a), *Mutpuracinus archibaldi* differs from Dasyuridae in: elongate snout; retention of three premolars; overall reduction of stylar crest and stylar cusps; reduction of paracones; widened angle of the centrocrista; reduction of protocones, conules, and distal cingulae; presence of a carnassial notch in the cristid obliqua (referred to as the prehypocristid by Murray & Megirian (2006a); uniform reduction of the metaconids; reduction of the entoconid. Differs from *Badjcinus turnbulli, Nimbacinus dicksoni*, and *Thylacinus cynocephalus* in: full enclosure of the petrosal and alisphenoid hypotympanic sinuses by the alisphenoid tympanic wing; posterior process of the maxillae narrow unlike *Thylacinus cynocephalus* in which the posterior maxillary processes flare widely, interposing the lacrimals, and nasals.

**Remarks:** The taxon is represented by several cranial specimens, including a near-complete cranium and partial mandible (Murray & Megirian, 2000, 2006a). Our findings agree with those of Archer et al. (2016), Kealy & Beck (2017), and Travouillon & Phillips (2018) in consistently falling outside Thylacinidae. The placement of *Mutpuracinus archibaldi* is a currently unresolved position within, sister, or stem to either Myrmecobiidae or Dasyuridae. We therefore refer *Mutpuracinus archibaldi* to Dasyuromorphia incertae sedis pending more conclusive results.

**Family** Thylacinidae Bonaparte, 1838
**Genus** *Badjcinus* Muirhead & Wroe, 1998
***Badjcinus turnbulli*** Muirhead & Wroe, 1998

**Figure 5:**
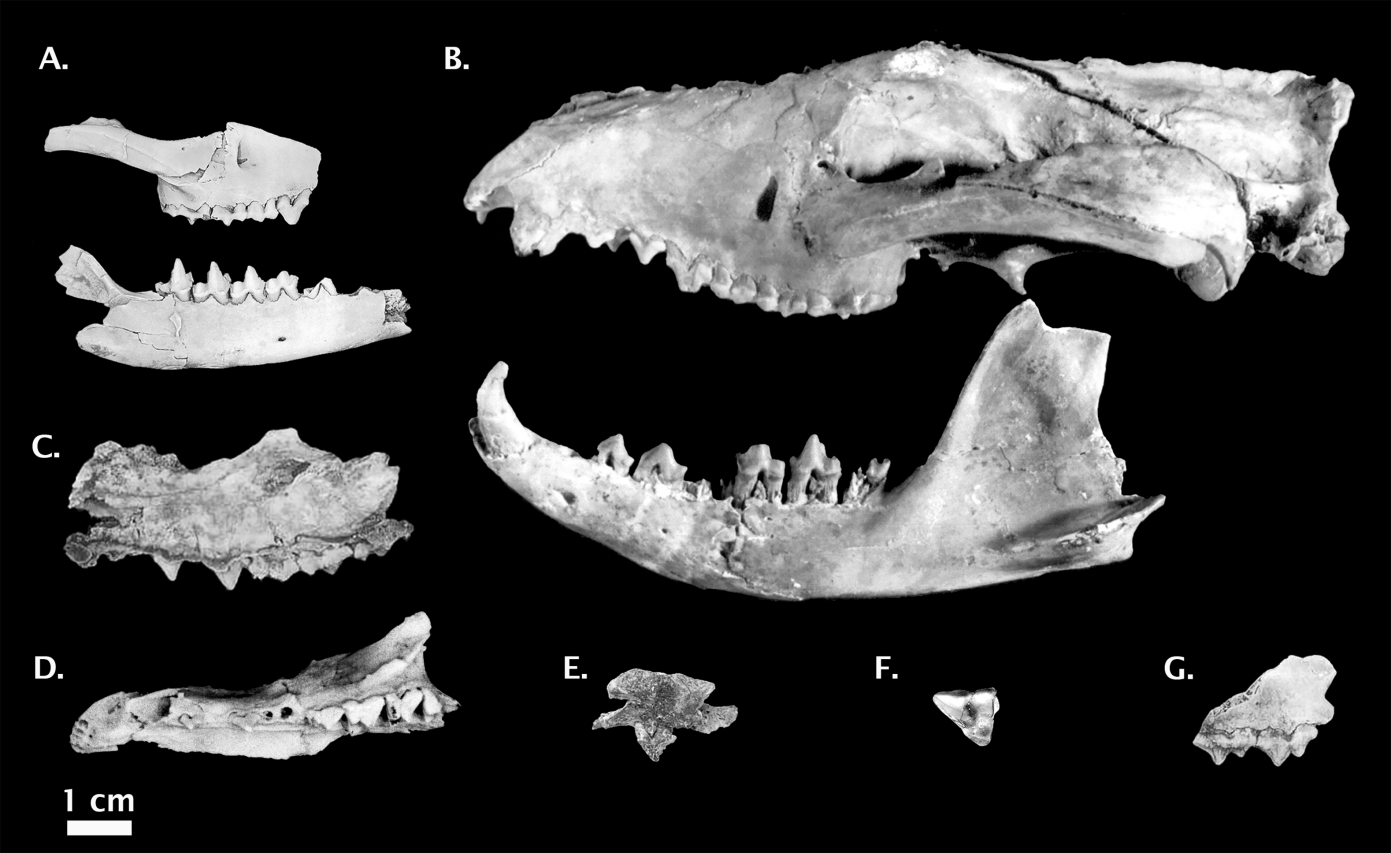
Non-*Thylacinus* fossil thylacinids. (A) *Muribacinus gadiyuli* QMF 30386 right maxilla and QMF 30385 right dentary. (B) *Nimbacinus dicksoni* QMF 36357 cranium and left dentary. (C) *Ngamalacinus timmulvaneyi* QMF 30300 left maxilla. (D) *Badjcinus turnbulli* QMF 30403 left premaxilla and maxilla. (E) *Tyarrpecinus rothi* NTM P98211 maxilla fragment. (F) *Maximucinus muirheadae* right M^2^. (G) *Wabulacinus ridei* QMF 16851 right maxilla fragment. Photo credits: (A) modified from Wroe (1996); (B) modified from Wroe & Musser (2001); (C) and (G) © Queensland Museum, Muirhead (1997); (D) modified from Wroe (2003); (E) modified from Murray & Megirian (2000), courtesy Museum and Art Gallery of the Northern Territory; (F) modified from Wroe (2001). All photos reproduced with permission.

**Holotype:** QM F30408, partial skull (Fig. 5D)

**Type Locality:** White Hunter Site, D-Site Plateau, Riversleigh World Heritage Area, Queensland, Australia

**Referred Specimens:** see Table S1

**Distribution and Age:** Riversleigh World Heritage Area, northwestern Queensland, Australia. The White Hunter Site is currently undated, but biocorrelation with the central Australian Ngama LF of South Australia suggests a late Oligocene date of ~26.0–24.0 Ma (Woodburne et al., 1994; Travouillon et al., 2006, 2011). However, fossil bats recovered from the site (*Brachipposideros nooraleebus*) have been noted to be similar to those found in the 17.4–16.8 Ma Bitesantennary Site as well as 20.4–16.0 Ma Burdigalian sites in France (Sigé, Hand & Archer, 1982; Archer et al., 1989; Hand & Archer, 2005). As such, the date is relatively uncertain, but the White Hunter site is likely to be latest Oligocene/earliest Miocene in age.

**Diagnosis:** Following Muirhead & Wroe (1998): very small thylacinid; lacking a squamosal epitympanic sinus; tympanic bulla lacks contribution by petrosal part of periotic; M^1^ preparacrista subparallel to the long axis of the tooth row; M_1_ and M_2–4_ metaconids differentially reduced. Distinguished from *Nimbacinus dicksoni* (Muirhead & Archer, 1990) by: stylar shelf, protocone, and conules reduced; more elongate postmetacristae; posterior cingulid joining hypocristid at base of hypoconid. Further differentiated from other similar-sized thylacinids by: protoconules and metaconules less reduced; entoconids and M_2–4_ metaconids relatively unreduced; hypocristid transversely oriented; cristid obliqua lacking carnassial notch and anterior termination less lingually shifted than in later occurring thylacinids.

**Remarks:** *Badjcinus turnbulli* is the earliest named thylacinid currently known, and is established from relatively complete cranial material. The known elements consist of a partial skull, including the left premaxilla, partial left maxilla, nasals, frontals, zygomatic arches, parietals, and a well-preserved basicranium, as well as partial left and right dentaries and a dentary fragment consisting mostly of dentition and alveolar bone (Muirhead & Wroe, 1998). The entire postcanine mandibular dentition excepting P_1_ are represented within the collected specimens, while the postcanine maxillary row is present excepting P^3^.

The rather plesiomorphic state of many of the characters of *Badjcinus turnbulli* has resulted in some uncertainty regarding its phylogenetic placement. It has occasionally been found to fall outside the clade as a potential dasyurid (Wroe et al., 2000) or stem-dasyuromorphian (see discussion in Kealy & Beck, 2017). Most phylogenetic analyses, however, recover it as sister to the remainder of Thylacinidae (Muirhead & Wroe, 1998; Wroe & Musser, 2001; Murray & Megirian, 2006a; Kealy & Beck, 2017).

**Genus** *Maximucinus* Wroe, 2001
***Maximucinus muirheadae*** Wroe, 2001

**Holotype:** QM F30331, right M^2^ (Fig. 5F).

**Type Locality:** Ringtail Site, Riversleigh World Heritage Area, Queensland, Australia.

**Referred Specimens:** none

**Distribution and Age:** Riversleigh World Heritage Area, northwestern Queensland, Australia. The middle Miocene Ringtail Site has been radiometrically dated to ~14.2–12.9 Ma (Woodhead et al., 2016).

**Diagnosis:** Modified from Wroe (2001): mid-sized thylacinid; all following features referring to M^2^: stylar cusps B and D well-developed and laterally compressed; anterior cingulum is continuous with the preparacrista; very small protoconule and metaconule.

**Remarks:** *Maximucinus muirheadae* is represented by a single M^2^ (Wroe, 2001). The tooth is interestingly large given the middle Miocene age of the specimen. The molar shows a large reduction in size of the protoconule and metaconule, but considering the well-developed stylar cusps is clearly less specialised towards hypercarnivory than that of derived members of Thylacinidae.

**Genus** *Muribacinus* Wroe, 1996
***Muribacinus gadiyuli*** Wroe, 1996

**Holotype:** QM F30386, partial right maxilla and jugal (Fig. 5A).

**Type Locality:** Dwornamor L, Gag Site, Riversleigh World Heritage Area, Queensland, Australia.

**Referred Specimens:** see Table S1

**Distribution and Age:** Riversleigh World Heritage Area, northwestern Queensland, Australia. *Muribacinus gadiyuli* is known from both Gag Site and Henk’s Hollow Site, Riversleigh. Neither localities have direct dates available, however, Gag Site and Henk’s Hollow have been found to be biostratigraphically correlated with the middle Miocene AL90 and Ringtail Sites (Travouillon et al., 2006, 2011; Arena et al., 2015). These correlated sites give a date range of ~15.1–12.9 Ma (Woodhead et al., 2016).

**Diagnosis:** Following Wroe (1996): very small thylacinid; differs from other thylacinids by: greater separation between paracones and metacones; large protocones; unreduced stylar shelf; preparacrista long relative to postmetacrista on M^1–3^; P_3_ smaller than P_2_; M_1–4_ metaconids less reduced; relatively large talonids.

**Remarks:** The holotype and paratype consist of a right maxillary and jugal fragment, respectively, and an unassociated, mostly complete right dentary (Wroe, 1996). Both the maxillary and mandibular specimens retain the last premolar and full molar row. *Muribacinus* has plesiomorphically unreduced metaconids on all four lower molars, indicating a possible early reversal in dental trends towards hypercarnivory in the clade.

**Genus** *Nimbacinus* Muirhead & Archer, 1990
***Nimbacinus dicksoni*** Muirhead & Archer, 1990
Syn. *Nimbacinus richi* Murray & Megirian, 2000

**Holotype:** QM F16802, left M_1_.

**Type Locality:** Henk’s Hollow Site, Gag Plateau, Riversleigh World Heritage Area, Queensland, Australia.

**Referred Specimens:** see Table S1

**Distribution and Age:** Riversleigh World Heritage Area, northwestern Queensland, and Bullock Creek, Northern Territory, Australia; all middle Miocene. *Nimbacinus dicksoni* has been recovered from the Riversleigh Henk’s Hollow and AL90 Sites. Henk’s Hollow has not been directly dated, but the two sites have been biostratigraphically correlated with each other and AL90 has been radiometrically dated to 15.1–14.2 Ma (Arena et al., 2015; Woodhead et al., 2016). Bullock Creek in Northern Territory is currently undated, but has been biostratigraphically allied with the Camfieldian Land Mammal Age (17–12 Ma; Arena et al., 2015).

**Diagnosis:** From Muirhead & Archer (1990): small thylacinid; unreduced stylar shelf with prominent stylar cusps B and D in addition to small stylar cusps C and E on M^1–2^; retention of prominent protoconules and metaconules on M^1–3^; prominent protocristae; retention of small metaconids on all lower molars.

**Remarks:** Unlike the majority of fossil thylacinids, *Nimbacinus dicksoni* is represented by multiple specimens, including a beautifully preserved, near-complete skull and mandible and a near-complete but to date undescribed skeleton (Muirhead & Archer, 1990; Wroe & Musser, 2001; Fig. 5B). The rostrum exhibits little of the mediolateral pinching characteristic of *Thylacinus* and is relatively short, with only modest diastemata present in the premolar rows, although there is a diastema between the mandibular canine and the P_1_ in *Nimbacinus dicksoni* contra *Thylacinus cynocephalus*. The skull is more robustly constructed than that of *Thylacinus cynocephalus* (Attard et al., 2014). The dentition of *Nimbacinus dicksoni* is relatively plesiomorphic for the family, with a reduction in stylar cusps, stylar shelf, and paracone that is greater than that of dasyurids, but an unelongated postmetacrista. The mandibular dentition is similarly plesiomorphic; the metaconids are reduced but present, and the talonid basin and protocone are reduced over that of dasyurids but not derived to the level seen in *Thylacinus*.

The validity of *Nimbacinus richi* has been questioned on the grounds of potential intraspecific variation and ambiguous fossil preservation (Wroe & Musser, 2001). As per Murray & Megirian (2000), *Nimbacinus richi* differs from *Nimbacinus dicksoni* in the differential expression of metaconids and entoconids, with *Nimbacinus richi* displaying a reduced metaconid on M_1_, large, well-developed metaconids on M_2–4_, and large conical entoconids on M_1–3_. The recovery of additional *Nimbacinus dicksoni* material (QM F36357) led Wroe & Musser (2001) to conclude that the differential metaconid expression shown between the putative specimens of *Nimbacinus richi* and *Nimbacinus dicksoni* falls within the range of variation exhibited by other known thylacinids. Furthermore, the authors note that the difference in entoconid size alone is likely to not be a viable character to differentiate species, as shown by variable entoconid expression (including presence/absence) in dasyurids (Dickman et al., 1998; Crowther, Dickman & Lynam, 1999).

Along with these arguments explicitly provided in Wroe & Musser (2001), the specimens of *Nimbacinus richi* (NTM P9612-4, P98695-92, and P904-7) are of a similar estimated body size to *Nimbacinus dicksoni* (range of 2.9–6.6 vs. 3.9–6.8 kg, respectively; see Table S2). We consider the likelihood of two near-identical species occurring in a temporally and spatially restricted space to be small. We find it more parsimonious to refer the *Nimbacinus ‘richi*’ specimens (NTM P9612-4, P98695-92, and P904-7) to *Nimbacinus dicksoni* rather than split the genus into two species.

**Genus** *Ngamalacinus* Muirhead, 1997
***Ngamalacinus timmulvaneyi*** Muirhead, 1997

**Holotype:** QM F16853, partial right dentary

**Type Locality:** Inabeyance Site, Godthelp Hill, Riversleigh World Heritage Area, Queensland, Australia

**Referred Specimens:** see Table S1

**Distribution and Age:** Riversleigh World Heritage Area, northwestern Queensland, Australia. *Ngamalacinus timmulvaneyi* is found at both the Inabeyance and Camel Sputum sites at Riversleigh (Muirhead, 1997). The Camel Sputum Site has been radiometrically dated to ~18.5–17.0 Ma (Woodhead et al., 2016). The age of the Inabeyance Site has not been directly dated, but biocorrelation with the RSO and Neville’s Garden Sites suggest an age of ~18.5–16.2 Ma (Arena et al., 2015; Woodhead et al., 2016).

**Diagnosis:** Modified from Muirhead (1997): small-sized thylacinid; relatively reduced conules and stylar shelf; retention of small stylar cusps B and D; narrower angle of maxillary molar cristae, less anteroposterior molar elongation, and less reduced talon basin than more derived thylacinids; retention of hypoconulid, entoconid, and relatively large metaconid (larger than paraconid) with distinct metacristid.

**Remarks:** The small thylacinid *Ngamalacinus timmulvaneyi* is represented by unassociated partial right dentary preserving M_1–4_, left maxilla preserving P^2^–M^3^, and an isolated M^2^ (Muirhead, 1997; Fig. 5C). The mandibular M_4_ is only partially erupted, indicating that this dentary belongs to a juvenile. *Nimbacinus timmulvaneyi* displays a mixture of plesiomorphic and derived dental characters without obvious specialisations towards hypercarnivory. There are, however, a handful of interesting characters to note. The maxillary dentition is moderately specialised, with relatively reduced stylar cusps and a reduction of the stylar shelf. The mandibular dentition is relatively plesiomorphic, with a relatively large talonid and possessing tall, distinct metaconids. The lower carnassial (M_4_), however, has a relatively reduced talonid basin with a present but small entoconid and hypoconid.

**Genus** *Thylacinus* Temminck, 1824
***Thylacinus macknessi*** Muirhead, 1992

**Figure 6:**
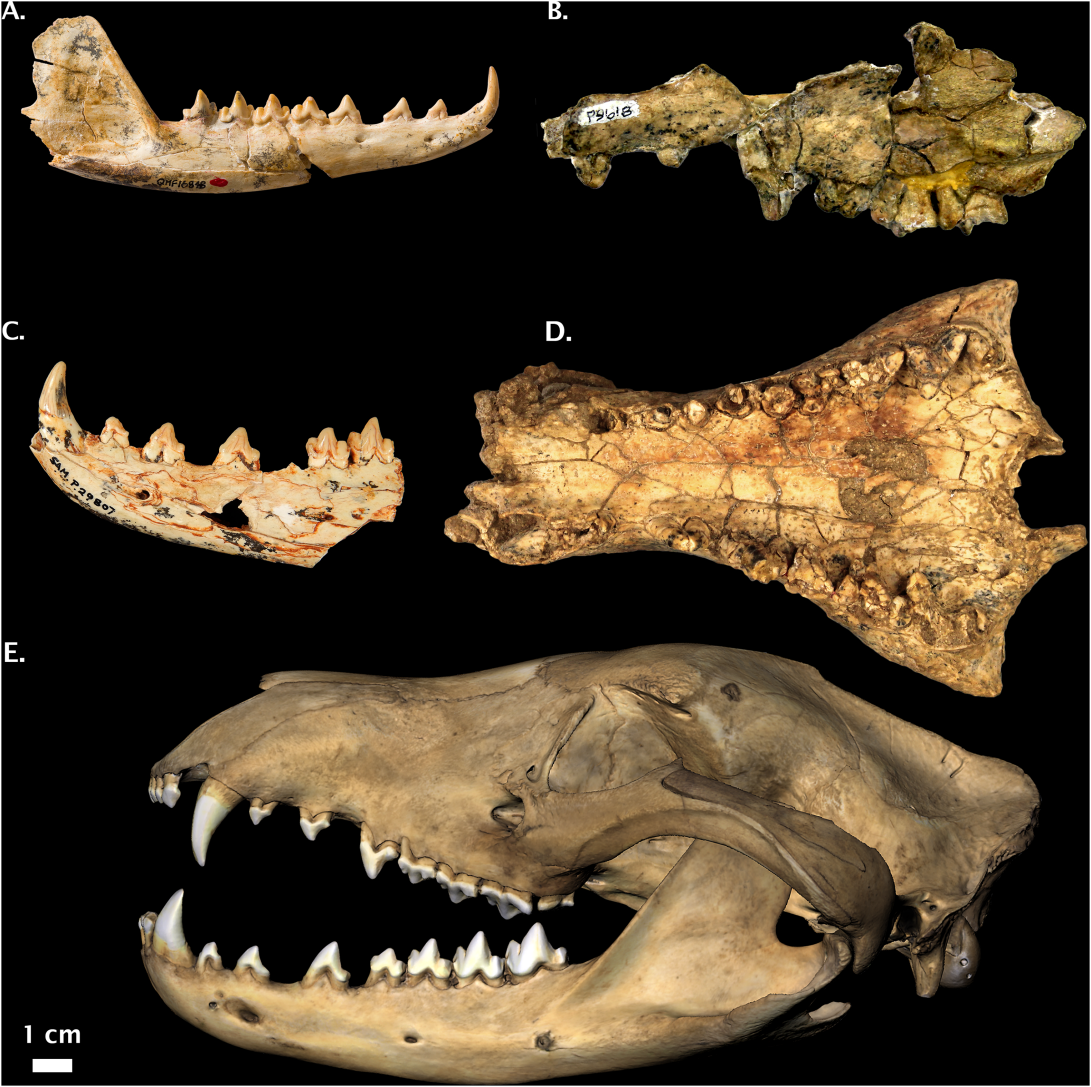
Non-Pleistocene fossil *Thylacinus* spp. (A) *Thylacinus macknessi* QMF 16848 right dentary. (B) *Thylacinus megiriani* NTM P9618 partial left maxilla. (C) *Thylacinus yorkellus* SAM P29807 partial left dentary. (D) *Thylacinus potens* CPC 6746 palatal view. (E) Modern *Thylacinus cynocephalus* WAM M195 3D surface scan for comparison (image reversed due to damage). Image credits: (A) © Queensland; (B) and (D): Museum and Art Gallery Northern Territory, Adam Yates; (C): © The Museum Board of South Australia, Mary-Anne Binnie; (E) DS Rovinsky. All photos reproduced with permission.

**Holotype:** QM F16848, right dentary (Fig. 6A).

**Type Locality:** Neville’s Garden Site, Riversleigh World Heritage Area, Queensland, Australia.

**Referred Specimens:** see Table S1

**Distribution and Age:** *Thylacinus macknessi* has been recovered from the early Miocene Neville’s Garden and Mike’s Menagerie sites, Riversleigh (Muirhead, 1992). Neville’s Garden has been dated to ~18.5–17.7 Ma, and Mike’s Menagerie estimated to ~18.5–16.2 via biostratigraphic correlation (Arena et al., 2015; Woodhead et al., 2016).

**Diagnosis:** Modified from the amended diagnosis by Muirhead & Gillespie (1995): a mid-sized thylacinid; M^1^ anterior cingulum well developed, continuous with protocrista, and lacking sulcus for preceding premolar; retention of small metaconule and lack of stylar shelf on M^1^; M^1^ with relatively unreduced paracone; retention of entoconid on all mandibular molars and a small metaconid on M_3–4_; M_1_ protoconid centrally located on crown, with the preprotocristid, postprotocristid, and cristid obliqua in line anteroposteriorly; reduction of anterior cingulum present on M_1_; main cusps of P_1–2_ anteriorly inclined and vertical on P_3_; anterior cuspule retained on P_1–3_; M_4_ anteroposteriorly shorter in length than preceding molar unlike other species of *Thylacinus*; coronoid process of the mandibular ramus departs from the corpus at a more acute angle than *Thylacinus cynocephalus* (~120° vs. ~130°).

**Remarks:** *Thylacinus macknessi* is known from a near-complete dentary and scattered mandibular dentition. The taxon shows a degree of the facial elongation characteristic of the genus, as well as dental reduction and cristae alignment indicative of the dental trending towards hypercarnivory.

There is an additional specimen (QM F16850; partial right M^1^) ascribed to the species by Muirhead (1992). This specimen is recovered from the Dwornamor LF of Gag Site, Riversleigh, long noted to be middle Miocene in age based on biocorrelation with the ~15.1–14.2 Ma AL90 site (Archer et al., 1989; Arena et al., 2015; Woodhead et al., 2016). The specimen is currently the only maxillary specimen attributed to this species, thus not directly referable to the holotype, and it is not clear precisely why Muirhead (1992) attributed the tooth to *Thylacinus macknessi*. At the time of this initial publication, there were no lower dentitions known that would have occluded with an upper first molar (i.e., M_1–2_; of which M_1_ was unknown and M_2_ missing anterior to the protoconid). Without a mandibular occlusal surface to match, it is difficult to have confidence in the attribution of the maxillary specimen. In a subsequent publication, Muirhead & Gillespie (1995) provide an amended description of the holotype QM F16848 after the anterior section of the dentary was discovered, but they do not supply any additional commentary regarding the M^1^ (QM F16850). We feel that pending the recovery of additional specimens supporting the alignment of QM F16850 with *Thylacinus macknessi*, it conservatively should be removed and placed within Thylacinidae *incertae sedis*.

***Thylacinus megiriani*** Murray, 1997

**Holotype:** NTM P9618, partial left maxilla (Fig. 6C).

**Type Locality:** Ongeva LF, Alcoota Station, Northern Territory, Australia.

**Referred Specimens:** see Table S1

**Distribution and Age:** The Ongeva LF has not been directly dated, but various biostratigraphic studies have shown correlations with the nearby Alcoota LF as well as the Beaumaris LF, Victoria (Murray, Megirian & Wells, 1993; Megirian, Murray & Wells, 1996; Megirian et al., 2010; Rich, Darragh & Vickers-rich, 2003; Black et al., 2012). Strontium dating of the Beaumaris LF has provided an age of ~6.2–4.5 Ma for that formation (Dickinson & Wallace, 2009). The Ongeva LF is at least slightly younger than the stratigraphically lower Alcoota LF (Woodburne, 1967; Murray, Megirian & Wells, 1993). The zygomaturine *Kolopsis torus* is present at both the Alcoota and Ongeva LFs but not Beaumaris LF, and it has been noted that *K. yperus*, present in the Ongeva LF, shares close similarities with and may be synonymous with the Beaumaris LF *K. gilli* (Megirian, Murray & Wells, 1996). This suggests that the three deposits all may have formed within a span of a few million years at most, with the Alcoota LF the earliest and Beaumaris LF the latest (Murray, Megirian & Wells, 1993; Megirian, Murray & Wells, 1996; Rich, Darragh & Vickers-rich, 2003; Megirian et al., 2010). As a conservative estimate, we consider the Alcoota LF to likely span 8.5–5.5 Ma, and the Ongeva LF 7.5–4.5 Ma.

**Diagnosis:** Following the revised diagnosis by Yates (2015): very large thylacinid; small postcingulum between the metastyle and protocone of M^2^; M^3^ mesiodistally longer than wide; absence of a precingulum on M^1^ and M^3^; absence of a metaconule on all maxillary molars; M^3^ much longer than M^2^; reduction of the paracone; relative elongation of the postmetacrista; hypertrophied torus along the ventrobuccal margin of the dentary; diastema between P_3_ and M_1_. Further differs from *Thylacinus potens* in: P^1^ in line with P^2–3^ instead of obliquely oriented.

**Remarks:** *Thylacinus megiriani* is only known from a partial maxillary fragment and recently described partial dentary fragments (Murray, 1997; Yates, 2015; Fig. 6C). The specimens suggest at an animal larger than the modern species, and of roughly similar rostral length but greater posterior palatal width than that of *Thylacinus potens*. Body mass based on geometric similitude with *Thylacinus cynocephalus* has estimated *Thylacinus megiriani* to be approximately 57.3 kg (Wroe, 2001), though see discussion below. As with *Thylacinus macknessi*, the general dental complexity reduction and elongation of shearing cristae indicate an increasing trend towards hypercarnivory.

***Thylacinus potens*** Woodburne, 1967

**Holotype:** CPC 6746, partial palate (Fig. 6B).

**Type Locality:** Alcoota LF, Alcoota Station, Northern Territory, Australia.

**Referred Specimens:** see Table S1

**Distribution and Age:** The Alcoota LF is likely to have spanned from 8.5 to 5.5 Ma; see the above discussion regarding *Thylacinus megiriani* for details.

**Diagnosis:** Following the amended diagnosis by Yates (2014): large thylacinid; mesiodistal axis of P^1^ mesiobuccally oriented; M^1^ mesiodistally longer than wide; palatal fenestrae greatly reduced; absence of a diastema between P_1–2_; P_2_ longer than P_3_ and M_1_. Yates (2014) additionally notes that *Thylacinus potens* may be further distinguished from *Thylacinus cynocephalus* by: ventrally facing sulcus forming the ventral border of the root of the zygomatic arch on the maxilla; P^2^ longer than M^1^.

**Remarks:** *Thylacinus potens* was the first pre-Pleistocene thylacinid to be discovered. The taxon is known from craniodental material including a large palatal fragment, maxillary and dentary fragments, and scant postcrania (Woodburne, 1967; Yates, 2014). A striking aspect of *Thylacinus potens* is the size of taxon, which has been estimated at 38.7 kg (via geometric similitude with *Thylacinus cynocephalus*; Wroe, 2001), and 40.9–120.6 kg (Yates, 2014). While the upper estimate is clearly an outlier probably caused by the relative robusticity of the dentition (see comments in Yates, 2014), it is clearly still substantially larger than the average body mass commonly cited for Tasmanian *Thylacinus cynocephalus* (29.5 kg; Paddle, 2000).

***Thylacinus yorkellus*** Yates, 2015

**Holotype:** SAM P29807, partial left dentary (Fig. 6D).

**Type Locality:** Curramulka LF, Cora-Lynn Cave, South Australia, Australia.

**Referred Specimens:** see Table S1

**Distribution and Age:** The Curramulka LF of Cora-Lynn Cave has yet to be directly dated. Megirian et al. (2010) suggest it is within the Tirarian Australian Land Mammal Age (defined therein at ~5.0–3.0 Ma). They note, however, that there may be limited temporal and taxonomic separation between deposits of the oldest Tirarian and youngest Waitean (the Waitean is defined as 12.0–~5.0 Ma), with some taxa sharing close phyletic ties across the two ages. Several taxa present in the Curramulka LF support the younger age, such as the Zanclean and younger *Baringa* cf. *nelsonensis, Tropsodon* cf. *bowensis*, and *Protemnodon* cf. *chinchillaensis* (Megirian et al., 2010). Despite intensive excavation no rodent fossils have been recovered from the Curramulka LF, suggesting that the fauna predates the expansion of rodents into Australia (Pledge, 1992; Yates, 2015). The oldest definitive fossil evidence of rodents, at both Bluff Downs and the Chinchilla LF, occurs by ~4.5–3.6 Ma (Hand, 1984; Pledge, 1992; Tedford, Wells & Barghoorn, 1992; Mackness, Whitehead & McNamara, 2000; Turnbull, Lundelius & Archer, 2003; Ogg, 2012). This date is supported by molecular evidence of the radiation of Sahul rodents (5.5–5.1 Ma) and their estimated expansion into Australia (3.7–3.4 Ma, CI: 4.5–2.4 Ma; (Rowe et al., 2008). This suggests a range of ~5.3–3.6 Ma for the Curramulka LF.

**Diagnosis:** Following Yates (2015): large thylacinid; strongly developed precingulid terminating in a cuspidule on the mesiobuccal face of the paraconid of M_1–3_; small basal mesial cuspidule on P_2–3_; absence of metaconids on M_2_, M_3_, and presumably M_4_; diastemata between C_1_–M_1_; mesiodistal lengths of P_2_ and P_3_ both exceeding that of M_1_.

**Remarks:** *Thylacinus yorkellus* was originally described by Pledge (1992) as *Thylacinus* sp. in the early 1990s, with a subsequent find (SAM P38799 right M_3_) prompting specific designation (Yates, 2015). The taxon is represented by an incomplete dentary fragment and isolated lower molar, and appears to have been rather longirostral, with diastemata between the canine—P_3_, as well as larger premolars than *Thylacinus cynocephalus*.

***Thylacinus* sp. indet.**

**Referred Specimens:** see Table S1

**Distribution and Age:** Big Sink LF (New South Wales), Chinchilla LF (Queensland), Awe LF (Papua New Guinea). Neither Big Sink LF nor Chinchilla LF have been directly dated. However, the biostratigraphic correlation of Chinchilla LF with the geomagnetically dated Kanunka LF of the Tirarian Fm suggests a date of ~4.2–3.6 Ma for the Chinchilla LF (Tedford, Wells & Barghoorn, 1992; Ogg, 2012). The Big Sink LF has been noted to be Tirarian in age and correlate based on biostratigraphy with the Chinchilla LF (Dawson, Muirhead & Wroe, 1999; Mackness et al., 2000; Megirian et al., 2010), giving a similar age. The Awe LF of Papua New Guinea is younger, with a K/Ar radiometric date of 3.3–2.4 Ma (Hoch & Holm, 1986).

**Remarks:** There are a small number of isolated specimens that have been referred to *Thylacinus* sp. or the modern *Thylacinus cynocephalus* and are purportedly recovered from Pliocene sediments of Australia and New Guinea. The New Guinea specimen consists of a partial dental fragment and is thus otherwise uninformative (UCMP 107737 partial P_2_; Awe LF, Papua New Guinea; Plane, 1976). *Thylacinus* sp. has also been recovered from the Big Sink LF of NSW (Dawson, Muirhead & Wroe, 1999). This specimen (AM F69875; partial left dentary with M_4_ preserved) is dentally similar in size to both the modern species and *Thylacinus potens* UCMP 66206, and unfortunately too incomplete for an assessment of the mandibular corpus regarding relative size/robusticity. Furthermore, the size of the M_4_ of *Thylacinus yorkellus* is unknown, and only a highly incomplete M_4_ of *Thylacinus megiriani* is currently known, both of which also may temporally overlap with AM F69875 (Yates, 2015).

The specimens from the Chinchilla LF have had a rather more interesting history. The attribution both of the taxa and the locality of these specimens has been contentious, as many of the specimens have been noted to have poor collection data (Mackness et al., 2002). The fragmentary nature of the specimens further precludes easy comparison, and therefore it has been difficult to confidently ascribe the specimens to a specific taxon. In a review of the 4.2–3.6 Ma Chinchilla Downs LF specimens, Mackness et al. (2002) note that the Chinchilla Downs locality attribution of several specimens were due to curatorial errors, and the *Thylacinus cynocephalus* specimens were likely recovered from the middle Pleistocene (144–73 ka; Price et al., 2011) Darling Downs. Furthermore, they note that the *Thylacinus* specimen that does actually originate from the Chinchilla LF (WPC 4506) lack specific diagnosable characters, which is especially noteworthy as the similar-sized *Thylacinus yorkellus* has been described from late Miocene/early Pliocene deposits in South Australia (Pledge, 1992; Yates, 2015). Louys & Price (2015) offer a dissenting view and note that at least two specimens (QM F3741 and F9476) definitively originate from the Chinchilla LF and are directly attributable to *Thylacinus cynocephalus*. However, Louys & Price (2015) do not offer any justification for ascribing the specimens to the modern taxon, and the single figure presented (QM F9476 dentary fragment) is ambiguous regarding attributes that would enable a specific designation, especially considering that the poorly temporally-constrained *Thylacinus yorkellus, Thylacinus megiriani* and potentially *Thylacinus potens* may also have been present within this general time period. Additionally, while certainly not impossible, it is perhaps unlikely that the modern species had persisted over a ~4 million year span. Without specific evidence to align the specimens in question to *Thylacinus cynocephalus*, we feel it is most conservative to defer specific attribution of the Pliocene material until an in depth analysis can be made of these specimens, their provenance, and their curatorial history.

**Genus** *Tyarrpecinus* Murray & Megirian, 2000
***Tyarrpecinus rothi*** Murray & Megirian, 2000

**Holotype:** NTM P98211, partial left maxilla (Fig. 5E).

**Type Locality:** Alcoota LF, Alcoota Station, Northern Territory, Australia.

**Referred Specimens:** none

**Distribution and Age:** The Alcoota LF is likely to have spanned from 8.5 to 5.5 Ma; see the above discussion regarding *Thylacinus potens* for details.

**Diagnosis:** After Murray & Megirian (2000): M^1^ narrow and elongate, with centrocrista relatively straight in relation to similar sized thylacinids excepting *W. ridei* Muirhead, 1997; strong ectoflexus and relatively elongate metastylar wing on M^3^; paracone closer to and smaller than metacone than in similar sized thylacinids; retention of stylar cusp B and D, with stylar cusp B relatively reduced on M^3^; conules reduced in size and number.

**Remarks:** *Tyarrpecinus rothi* is known only from a highly fragmented partial maxilla and associated dental fragments; nevertheless, it has been noted from the reconstructed fragments that the taxon seems to exhibit greater expression of dental characteristics related to hypercarnivory than in more basal forms (Murray & Megirian, 2000). The maxillary molars are more elongate, with a slight reduction in the complexity and size of cusps and increase in the elongation of shearing crests. That said, the poor preservation and extremely fragmentary nature precludes confidence in any firm comparisons. Interestingly, it has been suggested that the specimen derives from a crocodile coprolite due to its fragmented condition, coating of calcite, and evidence of potential acid etching (Murray & Megirian, 2000).

**Genus** *Wabulacinus* Muirhead, 1997
***Wabulacinus ridei*** Muirhead, 1997

**Holotype:** QM F16851, partial right maxilla (Fig. 5G)

**Type Locality:** Camel Sputum Site, Godthelp Hill, Riversleigh World Heritage Area, Queensland, Australia

**Referred Specimens:** see Table S1

**Distribution and Age:** Riversleigh World Heritage Area, northwestern Queensland, Australia. The Camel Sputum Site has a radiometric date of ~18.5–17.0 Ma (Woodhead et al., 2016).

**Diagnosis:** From Muirhead (1997): small thylacinid; infraorbital foramen wholly enclosed by the maxilla and positioned anterior to M^1^; preparacrista and centrocrista of M^1^ subparallel; entoconid absent and hypoconulid enlarged. Further differs from similar-sized thylacinids by: lack of stylar cusps B, D, and extreme reduction of talon and protocone on M^1^; M^1^ lacks sulcus for preceding premolar; lack of stylar cusp B and reduced stylar cusp D on M^2^; M_3_ metaconid reduced, lack of diastemata between mandibular dentition.

**Remarks:** The taxon is represented by a right maxillary fragment containing M^1–2^ and a partial left dentary preserving the alveoli for P_1_–M_4_ but containing only a broken M_3_ (Muirhead, 1997). The teeth show a marked reduction in the robusticity or the presence of many cusps and styles, a reduced protocone, especially on M^1^, and a reorientation of the major maxillary cristae of both preserved molars closer to parallel to the long axis of the tooth row. The dentary is relatively short, lacking diastemata between the premolars. While the mandibular dentition is unfortunately only represented by a partially preserved M_3_, the relatively reduced talonid basin and conids suggest the lower dentition is similarly reduced.

**Thylacinidae *incertae sedis*** Muirhead & Archer, 1990
Syn. *Nimbacinus dicksoni*

**Specimen:** QM F16809 partial right M_2_.

**Type Locality:** D-Site, Riversleigh World Heritage Area, Queensland, Australia.

**Distribution and Age:** D-site at Riversleigh is currently undated, but has been suggested to be late Oligocene via biostratigraphic correlation (Travouillon et al., 2006; Arena et al., 2015).

**Remarks:** *Nimbacinus dicksoni* has purportedly been recovered from the late Oligocene D-Site, Riversleigh (Muirhead & Archer, 1990). The attribution of this specimen to *Nimbacinus dicksoni* has been contentious, as both the late Oligocene age of the deposit and the state of metaconid reduction differs from that of the holotype, and the fragmentary nature of the specimen precludes further comparison (see discussion in Murray & Megirian, 2000; Wroe & Musser, 2001). We agree with the arguments presented by Murray & Megirian (2000) and Wroe & Musser (2001) and the conservative placement of the specimen QM F16809 as a thylacinid of uncertain position.

**Thylacinidae *incertae sedis*** Murray & Megirian, 2006b

**Specimen:** NTM P2815-10, fragmentary right M^2^.

**Type Locality:** Pwerte Marnte Marnte LF, Northern Territory, Australia.

**Distribution and Age:** The Pwerte Marnte Marnte LF is currently undated, but biostratigraphic correlation with the Etadunna Formation B & C suggests a late Oligocene age of ~25 Ma or older (Woodburne et al., 1994; Murray & Megirian, 2006b).

**Diagnosis:** Murray & Megirian (2006b) note that the fragmentary tooth is likely to be thylacinid due to the low stylar cusp D, relatively reduced stylar shelf, elongated metastylar wing, and narrow talon. It is comparable in size and gross morphology to the smallest known thylacinid, *Muribacinus*, differing, however in possessing a broader metastylar wing and larger protoconule.

**Remarks:** The fragmentary nature of the tooth prohibits any pointed discussion regarding the attribution or implications of the specimen.

## Discussion

### Phylogeny of the Thylacinidae

**Figure 7:**
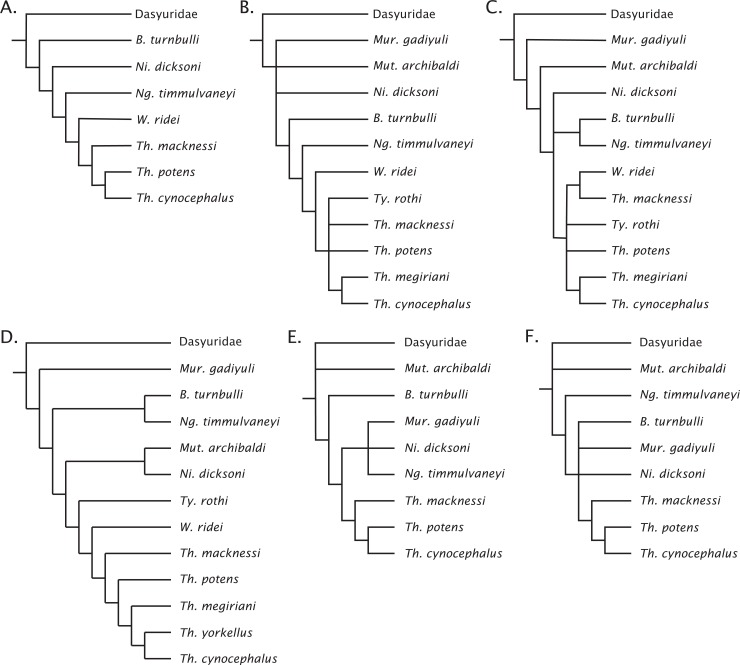
Previous concepts of intrafamily relationships in Thylacinidae. (A) After Muirhead (1997), Muirhead & Wroe (1998); (B) ****after Wroe (2003); (C) after Murray & Megirian (2000, 2006a); (D) after Yates (2014, 2015); (E) after Archer et al. (2016); (F) after Kealy & Beck (2017).

Although the fossil data set is comprised of 54% missing morphological characters, it recovers topologies across analyses that are consistent with those of prior studies (Fig. 7). The lack of resolution in the Bayesian morphological analysis along with the low support for the placements of the thylacinids in the tip-dated analysis does warrant caution. The current dataset is relatively small (less than 100 morphological characters) and there is no overlap across many taxa, even less so when taking the molecular data into consideration. The small size of the dataset potentially fails to offset this lack of overlap and missing data, which will tend to increase displacement of wildcard taxa (Wiens & Moen, 2008; Guillerme & Cooper, 2016). Additionally, the morphological dataset was compiled from previous studies that included only parsimony-informative characters, avoiding apomorphic characters. This can cause the loss of information regarding branch lengths and divergence times in Bayesian dating analyses (Lee & Palci, 2015; Dos Reis, Donoghue & Yang, 2016). Therefore, while these current results should be taken cautiously due to the current deficiencies of the analyses and the limitations of the published fossil thylacinid record, it is still encouraging to note that a consensus phylogenetic hypothesis is emerging.

### Feeding ecology

**Figure 8:**
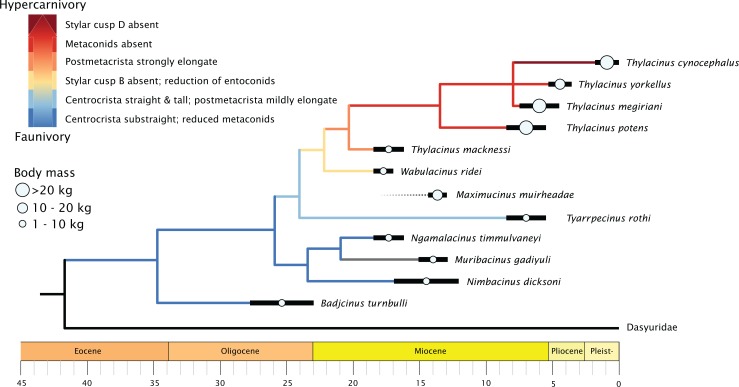
Time-scaled phylogeny of Thylacinidae, showing trends in body mass and increasing hypercarnivory. Tree is majority rules 50% consensus of 30 most parsimonious trees under implicit enumeration, time-scaled via ‘strap’ (Bell & Lloyd, 2014), with *Maximucinus muirheadae* added a posteriori. Body mass estimates compiled from Table S2.

When character state changes relating to the development of hypercarnivory are mapped to the timescale calibrated tree, a clear evolutionary trend is evident (Fig. 8). The family basally expresses a straightening of the centrocrista and reduction of metaconids, though with an apparent reversal seen in the retention of the M_1_ metaconid in the plesiomorphic *Muribacinus gadiyuli*. The clade inclusive of *W. ridei* + *Thylacinus cynocephalus* displays a marked shift towards the simplification of the molar dentition, as well as an elongation of the carnassial blade culminating in the hypercarnivorous condition seen in the terminal taxon.

During the initial period of thylacinid evolution (Oligocene through early Miocene) the group were thought to have occupied a niche broadly similar to that of the largest extant quoll (*Dasyurus maculatus*) or that of smaller extant canids, i.e., Vulpini (foxes) and Cerdocyonina (South American canids). Direct reconstruction of the feeding behaviour of the vast majority of the fossil thylacinids is hampered by the paucity of informative fossils, with most represented solely by cranial fragments and scattered dentition. There are, however, a small number with sufficiently informative remains to offer some degree of interpretation.

The plesiomorphic taxa *Badjcinus*, *Ngamalacinus*, and *Muribacinus* seem to have been rather unspecialised faunivores, lacking either the characteristics of more insectivorous dasyurids (e.g., unreduced stylar cusps, protocones, talonid basins, and metaconids) or characteristics of the more hypercarnivorous *Dasyurus maculatus* (e.g., robust protoconids, reduced metaconids, brachycephalisation). As Wroe (1996) suggested, this may have restricted the feeding niche of these members to invertebrate and smaller vertebrate prey. In contrast, biomechanical modelling of *Nimbacinus dicksoni* suggests that this taxon possessed a relatively robust cranial architecture, and was capable of taking prey items approaching or even exceeding its own mass (Attard et al., 2014), much like *Dasyurus maculatus* (Belcher, 1995).

Likewise, the craniodental characters of *W. ridei* suggest a similarly powerful hypercarnivore with the potential to take prey at or beyond its body size. The reduction of tooth complexity, the expansion in shearing crests, and the orientation of the cristae subparallel to the tooth row point to a high percentage of vertebrate flesh in the diet. The shorter dentary of the specimen suggests a relatively broad, stout rostrum, increasing masticatory efficiency and producing a more powerful bite, as well as increasing stress-handling ability when obtaining and processing prey items (e.g., see Radinsky, 1981; Greaves, 1983; Christiansen & Adolfssen, 2005; Tseng & Flynn, 2015).

The genus *Thylacinus* is marked by a strongly elongate postmetacrista, reduction or complete absence of metaconids, and increasing elongation of the rostrum (Fig. 8). Within the genus, *Thylacinus macknessi* retains a relatively short M_4_ and a small metaconid on all mandibular molars, in contrast to the lengthened carnassial and absent metaconids of later *Thylacinus*. This plesiomorphic condition suggests less specialisation towards hypercarnivory than seen in later members of the genus. *Thylacinus megiriani* is superficially similar in morphology to *Thylacinus cynocephalus*, albeit much larger and more robust, with slightly less elongate and simplified dentition. The dentition of the mid-sized *Thylacinus yorkellus* is also broadly similar to that of *Thylacinus cynocephalus*, though interestingly with relatively and absolutely larger premolars and slightly more robust molars.

The craniodental suite of *Thylacinus potens* is marked by more robust and less bladelike M^1–3^, an enlarged P^1–2^, greatly reduced (both relatively and absolutely) diastemata between P^1–3^, a greatly enlarged P_2_, no diastema between P_1–2_, and greatly reduced palatal fenestrae compared to *Thylacinus cynocephalus* (Woodburne, 1967; Murray, 1997; Yates, 2014). The result of this is a relatively shortened and reinforced rostrum with heavy, robust teeth. Additionally, the dentition of both the initial specimen and the subsequent discoveries of *Thylacinus potens* were noted to have heavily worn crowns (Woodburne, 1967; Yates, 2014). Yates (2014) has suggested that this combination of characters indicate what may be a degree of incipient durophagy—potentially carcass or bone processing. While this remains untested, it must be noted that *Thylacinus potens* does seem distinct within its radiation with respect to a combination of size, tooth attrition, and morphological characters that imply an increase in the handling ability of hard foodstuffs.

### Body mass and niche competition

Mass estimates for the larger-bodied *Thylacinus megiriani* and *Thylacinus potens* are potentially problematic, as they metrically sit outside the range of values used by Myers (2001) in the creation of mass estimation regression equations. As discussed by both Wroe (2001) and Yates (2014), these larger taxa also vary proportionally from the modern species, which may make the mass estimation via the equations of Myers (2001) inaccurate. To counter this, both Wroe (2001) and Yates (2014) employ the concept of geometric similitude with *Thylacinus cynocephalus* to estimate the mass of these larger taxa. It is unclear if this is necessarily a stronger approach, as if the taxa are too proportionally differentiated for an accurate estimation from the regression equations, it follows that they would be too proportionally differentiated for an accurate estimation from geometric similitude. Unfortunately, postcrania is virtually unknown for pre-Pleistocene thylacinids, with only a small handful of distal limb fragments described from *Thylacinus potens* (Woodburne, 1967) and a near-complete but as yet undescribed skeleton of *Nimbacinus dicksoni* (see Long et al., 2002; p.61). This leaves dental regression and geometric similitude as the best options for body mass estimation. Wroe (2001) found *Thylacinus megiriani* to weight approx. 57.3 kg and *Thylacinus potens* 38.7 kg, both by geometric similitude. *Thylacinus potens* was found by Yates (2014) to be an estimated 40.9–120.6 kg by a combination of geometric similitude and dental regression, with the noted caveat that the upper value is clearly an outlier potentially caused by the robust M^2^, leaving a far more plausible range of 40.9–56.0 kg. The mass estimates presented here in Table S2 for *Thylacinus megiriani* and *Thylacinus potens* (49.1 and 28.3–55.0 kg, respectively) generally agree with those found by both prior studies, and it can be expected that both taxa commonly approached >40 kg.

**Figure 9:**
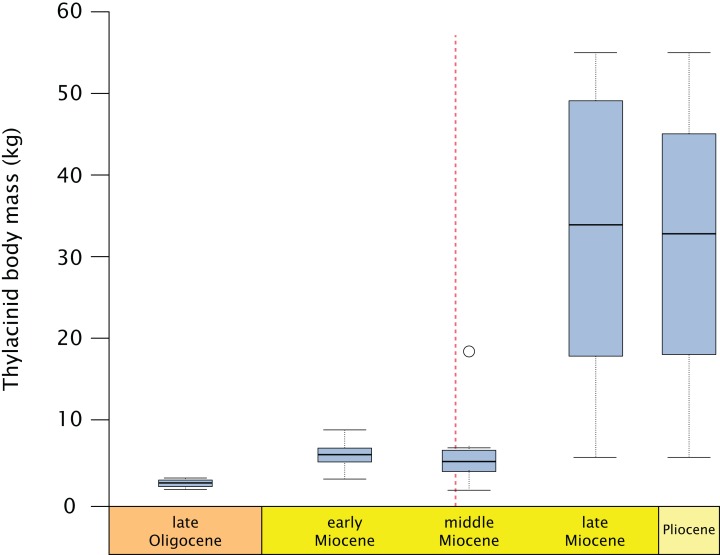
Fossil thylacinid body mass over time. All thylacinid specimens body mass estimates utilising Myers (2001) dasyuromorphian-only dental regressions (see Table S2), binned into the late Oligocene (= Chattian), late, middle, and early Miocene, and Pliocene. Dashed line = Middle Miocene Climate Transition (MMCT). Outlier in the middle Miocene is *Maximucinus muirheadae*.

Thylacinids were relatively small-bodied throughout the Oligocene and most of the Miocene. With the exception of *Maximucinus muirheadae* (known only from a single molar) every non-*Thylacinus* member of the clade has been estimated at well under 10.0 kg in mass (Table S2; Fig. 9). The dramatic shift in mass within *Thylacinus* coincides with the ~15–14 Ma middle Miocene climatic transition (MMCT), a period of dramatic global cooling and aridification, and the subsequent radiation of the Dasyuridae (Flower & Kennett, 1994; Crowther & Blacket, 2003; Wroe, 2003; Beck, 2008; Kealy & Beck, 2017; Eldridge et al., 2019). The MMCT therefore marks a point of both significant expansion in thylacinid body mass as well as a possible reduction in generic diversity, during a time of drastic shifts toward the aridification and cooling of the Australian environment and potentially rapid expansion of competitors.

A potential cause for this shift in the carnivorous marsupial guild structure during the MMCT has been suggested to be related to the differing morphology of the auditory systems of the taxa. The morphology of the thylacinid middle ear is very plesiomorphic, with a small petrosal contribution to the tympanic wing. This is in direct contrast with the dasyurid condition, in which the hypotympanic and epitympanic regions are fully enclosed by bony expansions of the tympanic processes (Wroe, 1997). Under this scenario, this has been suggested to have conferred a distinct advantage to the dasyurids over the thylacinids, improving their low-frequency hearing and perhaps facilitating the competitive exclusion of the latter taxa as aridification of the environment after the MMCT opened up what was previously forest (Wroe, 1996, 1997; Kealy & Beck, 2017). It has additionally been suggested that early peramelemorphians such as *Bulungu palara* and *Galadi speciosus* may have occupied the smaller-bodied (i.e., <1 kg) carnivore niche during the early Miocene and were, similarly to the thylacinids, replaced during the major faunal turnover event following the MMCT (Travouillon et al., 2010; Gurovich et al., 2014). Notably, both the thylacinids and the Oligo–Miocene peramelemorphians retained a relatively plesiomorphic middle ear condition without a full bony enclosure; this ostensibly would limit their low-frequency auditory sensitivity and place them at a competitive disadvantage with the dasyurids. The potential for ecologic displacement of two carnivorous lineages, a <1 kg peramelemorphian group and a <10 kg thylacinid group by the rapidly diversifying dasyurids, is intriguing. However, it is ultimately unknown whether it was competitive replacement by the dasyurids or their radiation into recently vacated niches emptied by the extinction of the peramelemorphian and small-bodied thylacinids that drove the change in guild structure.

### Biogeography

**Figure 10:**
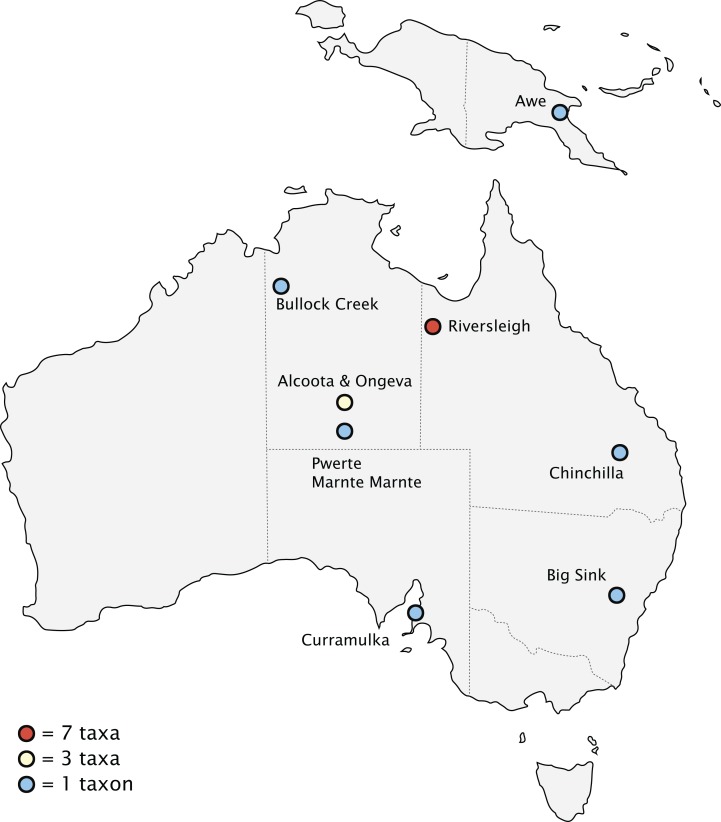
Pliocene and older (>2.58 Ma) thylacinid-bearing fossil sites. Site icons coded by minimal number of taxa recovered.

Fossils of thylacinids have been recovered from the Riversleigh World Heritage Fossil Site, Queensland, and the Bullock Creek and Alcoota/Ongeva localities, Northern Territory, with scattered specimens from additional localities along the eastern interior of the continent (Fig. 10). The well-sampled nature of the Riversleigh sites allows for a relatively secure interpretation of the palaeoenvironment during the early evolution of the family. The late Oligocene of the Australian interior seems to have been one of cool, wet, seasonal open forests, with a trend of increasing humidity, warmth, and widespread closed forest during the early Miocene through to the ~16 Ma middle Miocene climatic optimum (Flower & Kennett, 1994). These environmental conditions changed dramatically after the ~15–14 Ma MMCT. This led to a pronounced constriction and fragmentation of the wet forest environments and the dramatic expansion of sclerophyll vegetation communities. The late Miocene of Australia saw the transition to a subhumid-semiarid, highly seasonal environment, with the widespread overall replacement of the early and mid-Miocene wet rainforest with open-canopy sclerophyll forest and increasingly seasonal rainfall patterns (Black et al., 2012). This climatic shift continued through the Pliocene, with the first indication of widespread open grasslands along with the rapid diversification of grazing taxa appearing after 4 Ma (Dawson & Dawson, 2006; Strömberg, 2011).

It is tempting to speculate about what effect this environmental shift from wet forests to a dry, open sclerophyll community had on the basal ecology and evolutionary trends of the thylacinids. Comparison with the craniodental skeleton of dasyurids suggests a broadly similar dietary ecology, but the lack of thylacinid postcranial specimens leaves doubt as to their locomotor habits. It has been suggested that the modern thylacine expressed more derived cursorial morphology than other metatherians, though not as derived as their eutherian counterparts (Smith, 1982; Jones & Stoddart, 1998; Argot, 2004; Figueirido & Janis, 2011; Janis & Figueirido, 2014). This raises the question as to whether these earlier thylacinids were similarly cursorial-adapted like the modern species, or if they were scansorial like the dasyurids that replaced them. If early thylacinids were plesiomorphically scansorial, it is feasible that the dasyurid radiation pushed them out of the trees and onto the ground, favouring increasingly terrestrial adaptations (e.g., larger body size, cursorial locomotion).

Alternatively, this apparent ecological shift could simply be an artefact due to the very limited geographic sampling presented by the fossil localities. Except for *Nimbacinus dicksoni* and the indeterminate Pwerte Marnte Marnte specimen (NTM P2815-10), every middle-Miocene and older thylacinid is recovered from the Riversleigh deposits. All these thylacinids with the exception of the ~18.4 kg *Maximucinus muirheadae* are estimated at <10 kg. It is possible that larger-bodied terrestrial thylacinids were present and penecontemporaneous with the smaller forms outside the rainforests of Riversleigh. The Oligocene through early Miocene deposits of central and Western Australia differed notably from the concurrent Riversleigh deposits. The central Australian environment was marked by alkaline lakes, ephemeral swamps, and sclerophyll communities, and Western Australia by *Eucalyptus* and Casuarinaceae communities (Martin, 2006). This suggests a stronger seasonality and a more arid, open environment than the Riversleigh deposits, though both environments were significantly wetter than in the modern day. We do find the large Mio-Pliocene *Thylacinus potens* and *Thylacinus megiriani* in the Northern Territory Alcoota and Ongeva LFs, both thought to have been seasonal fluvio-lacustrine or waterhole deposits in a relatively semi-arid, more open environment than the ‘classic’ forested Riversleigh deposits (Murray & Megirian, 1992; Megirian, Murray & Wells, 1996). However, these sites likely post-date the MMCT by at least 5 million years, prohibiting any conclusion regarding the life habits of the early thylacinids.

## Conclusions

The fossil history of the Thylacinidae can be seen as a series of steps toward hypercarnivory, coupled with a marked shift in body mass after the Middle Miocene Climate Transition. As has been suggested in prior studies (Crowther & Blacket, 2003; Wroe, 2003; Kealy & Beck, 2017), it is possible that these shifts, particularly the dramatic shift in body mass, may have occurred in response to the selective pressure created by the emergence of the dasyurids. The radiation of dasyurids appears to have replaced both the thylacinids and the peramelemorphians in the >10 kg carnivore niche following the MMCT. Whether this was due to competitive replacement or simply filling the niches left vacant by the extinction of these small-bodied thylacinid and peramelemorphian carnivores is uncertain. As the Australian Cenozoic fossil record expands through excavation, description, and analysis these gaps in thylacinid evolutionary history may be more confidently addressed.

The pre-Pleistocene fossil thylacinids provide an evolutionary framework for understanding the modern taxon. Integration of data regarding the timing and trends in increasing body mass, hypercarnivory, and cursoriality may allow future studies to build a clearer understanding of the niche ecology of the thylacine. This would help to avoid spurious conclusions regarding convergence, or conversely, to help identify and quantify degree of convergence through time.

Comparing the evolution of hypercarnivory and large body size of the non-marsupial metatherian South American borhyaenids and other sparassodonts (e.g., Hathliacynidae) with the thylacinids would be an intriguing future study. Incorporating both of these carnivorous metatherian groups, the thylacinids and sparassodonts, with potential eutherian analogues within Canidae would also be highly informative. These comparisons may help researchers understand the extent of morphological and ecological convergence across carnivorous mammals through evolutionary time, and may ground further research into the disparate groups. Furthermore, as Bayesian analyses become more prevalent, the scoring of apomorphic characters for both fossil and extant animals will greatly facilitate phylogenetic analyses and our understanding of the evolution of these groups. These future studies will ultimately depend upon further, more complete fossil thylacinid specimens and the discoveries of new sites filling critical gaps in the Australian fossil record.

## Supplemental Information

10.7717/peerj.7457/supp-1 Supplemental Information 1 Published non-Pleistocene fossil Thylacinidae specimens.

10.7717/peerj.7457/supp-2 Supplemental Information 2 Body mass estimates of fossil Thylacinidae. Mass estimated using Dasyuromorphia-only regressions from Myers (2001). Estimate includes smearing factor. SEE%, percent standard error of the estimate; PE%, percent prediction error; SE%, smearing estimate. All mass estimates from current study except ^1Wroe (2001)^ and ^2Yates (2014)^.

10.7717/peerj.7457/supp-3 Supplemental Information 3 Genbank Accession numbers.

10.7717/peerj.7457/supp-4 Supplemental Information 4 Parsimony TNT file. Morphology-only parsimony analysis. Includes data and instructions to force *Mutpuracinus* onto the Thylacinidae branch.

10.7717/peerj.7457/supp-5 Supplemental Information 5 MrBayes morphology NEXUS file.

10.7717/peerj.7457/supp-6 Supplemental Information 6 MrBayes tipdate NEXUS file.

10.7717/peerj.7457/supp-7 Supplemental Information 7 Fossil date ranges and Newick trees for Strap analyses.

10.7717/peerj.7457/supp-8 Supplemental Information 8 Character list. Modified from matrices presented in Yates (2014) and Kealy & Beck (2017).

10.7717/peerj.7457/supp-9 Supplemental Information 9 R-code for Strap time-scale analysis.

10.7717/peerj.7457/supp-10 Supplemental Information 10 Templeton test TNT file.

10.7717/peerj.7457/supp-11 Supplemental Information 11 Tree node age estimates. (A) Age estimate for the parsimony analysis. Tree generated by implicit enumeration in TNT and fed to strap along with FAD/LAD of taxa. Numbers are years (Ma). Node age estimate comparison for strap and Bayesian FBD analyses. Tree generated by Bayesian tip-dated FBD analysis and fed to strap along with FAD/LAD of taxa. Numbers at nodes are node ages (Ma), top (purple) is strap, bottom (black) is Bayesian. Data in Dataset S4, R code in File S2.

## INSTITUTIONAL ABBREVIATIONS

AM: Australian Museum (Sydney)
CPC: Commonwealth Palaeontological Collection (Canberra)
NTM: Northern Territory Museum (Darwin and Alice Springs)
QM: Queensland Museum (Brisbane)
SAM: South Australian Museum (Adelaide)
UCMP: Museum of Paleontology, University of California (Berkeley)
WAM: Western Australian Museum
WPC: Wilkinson Private Collection (Chinchilla).

## Additional Information and Declarations

### Data Availability

The following information was supplied regarding data availability: Specimen list are available in Table S1. Specimens are stored at: Australian Museum, Sydney (AM F69875); Commonwealth Palaeontological Collection, Canberra (CPC 6746); Northern Territory Museum and Art Gallery, Alice Springs (NTM P2815-10, P4326, P4327, P4332, P4376, P4377, P4379, P4461, P4516, P85553-3, P8695-92, P87108-10, P904-7, P907-3, P91168-5, P9464-119, P9464-120, P9612-4, P9612-5, P9618, P98211); Queensland Museum, Brisbane (QM F16802, F16803, F16804, F16805, F16806, F16807, F16809, F16848, F16848b, F16850, F16851, F16852, F16853, F16855, F19849, F30300, F30331, F30385, F30386, F30407, F30408, F30409, F30410, F30411, F36357, F3741, F9476); South Australian Museum, Adelaide (SAM P29807, P38799); Museum of Paleontology, Berkeley (UMCP 107737, 66199, 66206, 66649, 66971, 69657, 69658, 69659, 69677, 71011, 71012); Western Australian Museum, Perth (WAM M195); and Wilkinson Private Collection, Chinchilla (WPC 4506). Body mass data are available in Table S2. Genbank numbers are listed in Table S3. Matrices for the phylogenetic analyses are available in Datasets S1–S4. Fossil dasyurid dates are also available in Dataset S4. Character list and descriptions are available in File S1. R code for ‘strap’ time-scaling is available in File S2. Templeton test data available in File S3. Node date estimation are available in Fig. S1.
