# Supplementary material for: The pre-Pleistocene fossil thylacinids (Dasyuromorphia: Thylacinidae) and the evolutionary context of the modern thylacine
Source: PeerJ. 2019 Sep 2;7:e7457. doi: 10.7717/peerj.7457 (PMC6727838; doi:10.7717/peerj.7457)
Supplement: Supplemental Information 1 [file peerj-07-7457-s001.docx]

| Specimen | Taxon | Material | Locality | Age (Ma) | Reference(s) |
| --- | --- | --- | --- | --- | --- |
| QM F30408 | *Badjcinus turnbulli* | Partial skull (Left: P^2-3^; M^1-4^) | White Hunter Site, D-Site Plateau, Riversleigh QLD | Late Oligocene  (>23.0) | Muirhead & Wroe, 1998 |
| QM F30409 | *Badjcinus turnbulli* | Right M_1_ | White Hunter Site, D-Site Plateau, Riversleigh QLD | Late Oligocene  (>23.0) | Muirhead & Wroe, 1998 |
| QM F30407 | *Badjcinus turnbulli* | Partial right dentary (M_1-4_) | White Hunter Site, D-Site Plateau, Riversleigh QLD | Late Oligocene  (>23.0) | Muirhead & Wroe, 1998 |
| QM F30410 | *Badjcinus turnbulli* | Partial left dentary (P_2_; M_1-4_) | White Hunter Site, D-Site Plateau, Riversleigh QLD | Late Oligocene  (>23.0) | Muirhead & Wroe, 1998 |
| QM F30411 | *Badjcinus turnbulli* | Partial right dentary (P_2-3_; M_1-3_) | White Hunter Site, D-Site Plateau, Riversleigh QLD | Late Oligocene  (>23.0) | Muirhead & Wroe, 1998 |
| QM F30331 | *Maximucinus muirheadae* | Right M^2^ | Ringtail Site, Riversleigh, QLD | Middle Miocene  (14.2-12.9) | Wroe, 2001 |
| QM F30386 | *Muribacinus gadiyuli* | Partial right maxilla & jugal (P^3^; M^1-4^) | Gag Site, Riversleigh, QLD | Middle Miocene  (15.1-14.2) | Wroe, 1996 |
| QM F30385 | *Muribacinus gadiyuli* | Partial right dentary (P_3_-M_4_) | Henk's Hollow Site, Riversleigh, QLD | Middle Miocene  (15.1-12.9) | Wroe, 1996 |
| QM F16853 | *Ngamalacinus timmulvaneyi* | Partial right dentary (M_1-4_) | Inabeyance Site, Godthelp Hill, Riversleigh QLD | Early Miocene  (18.5-16.2) | Muirhead, 1997 |
| QM F30300 | *Ngamalacinus timmulvaneyi* | Left maxilla (P^2^-M^3^) | Camel Sputum Site, Godthelp Hill, Riversleigh QLD | Early Miocene  (18.5-17.0) | Muirhead, 1997 |
| QM F16855 | *Ngamalacinus timmulvaneyi* | Right M^2^ | Inabeyance Site, Godthelp Hill, Riversleigh QLD | Early Miocene  (18.5-16.2) | Muirhead, 1997 |
| QM F16802 | *Nimbacinus dicksoni* | Left M_1_ | Henk's Hollow Site, Riversleigh, QLD | Middle Miocene  (15.1-12.9) | Muirhead & Archer, 1990 |
| QM F16803 | *Nimbacinus dicksoni* | Partial Right maxilla (P^3^-M^3^) | Henk's Hollow Site, Riversleigh, QLD | Middle Miocene  (15.1-12.9) | Muirhead & Archer, 1990 |
| QM F16804 | *Nimbacinus dicksoni* | Partial right maxilla (M^1-3^) | Henk's Hollow Site, Riversleigh, QLD | Middle Miocene  (15.1-12.9) | Muirhead & Archer, 1990 |
| QM F16805 | *Nimbacinus dicksoni* | M^3^ | Henk's Hollow Site, Riversleigh, QLD | Middle Miocene  (15.1-12.9) | Muirhead & Archer, 1990 |
| QM F16806 | *Nimbacinus dicksoni* | M^3^ | Henk's Hollow Site, Riversleigh, QLD | Middle Miocene  (15.1-12.9) | Muirhead & Archer, 1990 |
| QM F16807 | *Nimbacinus dicksoni* | M^4^ | Henk's Hollow Site, Riversleigh, QLD | Middle Miocene  (15.1-12.9) | Muirhead & Archer, 1990 |
| NTM P85553-3 | *Nimbacinus dicksoni* | Partial right dentary (P_1-2_; M_1_) | Bullock Creek, NT | Middle Miocene  (17-12) | Muirhead & Archer, 1990 |
| QM F36357 | *Nimbacinus dicksoni* | Skull & mandible | AL90 Site, Riversleigh, QLD | Middle Miocene  (15.1-14.2) | Wroe & Musser, 2001 |
| NTM P9612-4 | Nimbacinus dicksoni (syn. *Nimbacinus richi* Murray & Megirian, 2000) | Right dentary (P_1_-M_4_) | Top Site, Bullock Creek, NT | Middle Miocene  (17-12) | Murray & Megirian, 2000 |
| NTM P8695-92 | Nimbacinus dicksoni (syn. *Nimbacinus richi* Murray & Megirian, 2000) | Partial left dentary (P_2_, M_1_, M_3-4_) | Blast Site, Bullock Creek, NT | Middle Miocene  (17-12) | Murray & Megirian, 2000 |
| NTM P904-7 | Nimbacinus dicksoni (syn. *Nimbacinus richi* Murray & Megirian, 2000) | Partial left dentary (M_2-4_) | Top Site, Bullock Creek, NT | Middle Miocene  (17-12) | Murray & Megirian, 2000 |
| QM F16848 | *Thylacinus macknessi* | Right dentary (C_1_-M_4_) | Neville's Garden Site, Riversleigh, QLD | Early Miocene  (18.5-17.7) | Muirhead, 1992; Muirhead & Gillespie, 1995 |
| QM F16848b | *Thylacinus macknessi* | Right C_1_ | Neville's Garden Site, Riversleigh, QLD | Early Miocene  (18.5-17.7) | Muirhead, 1992 |
| QM F19849 | *Thylacinus macknessi* | Left M_3_ | Mike's Menagerie Site, Godthelp Hill, Riversleigh, QLD | Early Miocene  (18.5-16.2) | Muirhead, 1992 |
| NTM P9618 | *Thylacinus megiriani* | Partial left maxilla (P^1^; P^3^; M^1-4^) | Ongeva Local Fauna, Alcoota Station, NT | Late Miocene - early Pliocene (7.5-4.5) | Murray, 1997 |
| NTM P4376 | *Thylacinus megiriani* | Partial right dentary | Ongeva Local Fauna, Alcoota Station, NT | Late Miocene - early Pliocene (7.5-4.5) | Yates, 2015 |
| NTM P4377 | *Thylacinus megiriani* | Partial right dentary (M_4_ fragmentary) | Ongeva Local Fauna, Alcoota Station, NT | Late Miocene - early Pliocene (7.5-4.5) | Yates, 2015 |
| CPC 6746 | *Thylacinus potens* | Partial palate (left M^2-4^; right P^2^-M^2^) | Alcoota Local Fauna, Alcoota Station, NT | Late Miocene - early Pliocene (8.5-5.5) | Woodburne, 1967 |
| UCMP 66971 | *Thylacinus potens* | Left M^3^ | Alcoota Local Fauna, Alcoota Station, NT | Late Miocene - early Pliocene (8.5-5.5) | Woodburne, 1967 |
| UCMP 71012 | *Thylacinus potens* | Left M^4^ fragment | Alcoota Local Fauna, Alcoota Station, NT | Late Miocene - early Pliocene (8.5-5.5) | Woodburne, 1967 |
| UCMP 66199 | *Thylacinus potens* | Left C_1_ | Alcoota Local Fauna, Alcoota Station, NT | Late Miocene - early Pliocene (8.5-5.5) | Woodburne, 1967 |
| UCMP 66206 | *Thylacinus potens* | Partial left dentary (M_2-4_) | Alcoota Local Fauna, Alcoota Station, NT | Late Miocene - early Pliocene (8.5-5.5) | Woodburne, 1967 |
| UCMP 71011 | *Thylacinus potens* | Partial left dentary (P_3_-M_1_) | Alcoota Local Fauna, Alcoota Station, NT | Late Miocene - early Pliocene (8.5-5.5) | Woodburne, 1967 |
| UCMP 66649 | *Thylacinus potens* | Left calcaneum | Alcoota Local Fauna, Alcoota Station, NT | Late Miocene - early Pliocene (8.5-5.5) | Woodburne, 1967 |
| UCMP 69677 | *Thylacinus potens* | Left astragalus | Alcoota Local Fauna, Alcoota Station, NT | Late Miocene - early Pliocene (8.5-5.5) | Woodburne, 1967 |
| UCMP 69657 | *Thylacinus potens* | Partial left metatarsal 4 | Alcoota Local Fauna, Alcoota Station, NT | Late Miocene - early Pliocene (8.5-5.5) | Woodburne, 1967 |
| UCMP 69658 | *Thylacinus potens* | Right metatarsal 4 | Alcoota Local Fauna, Alcoota Station, NT | Late Miocene - early Pliocene (8.5-5.5) | Woodburne, 1967 |
| UCMP 69659 | *Thylacinus potens* | Partial right metatarsal 3 | Alcoota Local Fauna, Alcoota Station, NT | Late Miocene - early Pliocene (8.5-5.5) | Woodburne, 1967 |
| NTM P4326 | *Thylacinus potens* | Partial right maxilla (P^2-3^; M^2-4^) | Alcoota Local Fauna, Alcoota Station, NT | Late Miocene - early Pliocene (8.5-5.5) | Yates, 2014 |
| NTM P4327 | *Thylacinus potens* | Partial left dentary (P_2-3_; M_1-4_) | Alcoota Local Fauna, Alcoota Station, NT | Late Miocene - early Pliocene (8.5-5.5) | Yates, 2014 |
| NTM P4332 | *Thylacinus potens* | Left P^3^ | Alcoota Local Fauna, Alcoota Station, NT | Late Miocene - early Pliocene (8.5-5.5) | Yates, 2014 |
| NTM P4379 | *Thylacinus potens* | Partial maxilla (M^2^) | Alcoota Local Fauna, Alcoota Station, NT | Late Miocene - early Pliocene (8.5-5.5) | Yates, 2014 |
| NTM P4461 | *Thylacinus potens* | Right C_1_ | Alcoota Local Fauna, Alcoota Station, NT | Late Miocene - early Pliocene (8.5-5.5) | Yates, 2014 |
| NTM P4516 | *Thylacinus potens* | Right maxillary molar fragment | Alcoota Local Fauna, Alcoota Station, NT | Late Miocene - early Pliocene (8.5-5.5) | Yates, 2014 |
| QM F3741 | *Thylacinus* sp. | Partial right maxilla | Chinchilla Local Fauna, QLD | Late Pliocene (4.2-3.6) | Louys & Price, 2015 |
| QM F9476 | *Thylacinus* sp. | Partial right dentary (M_4_) | Chinchilla Local Fauna, QLD | Late Pliocene (4.2-3.6) | Louys & Price, 2015 |
| WPC 4506 | *Thylacinus* sp. | Partial right dentary (M_2-4_) | Chinchilla Local Fauna, QLD | Late Pliocene (4.2-3.6) | Mackness et al., 2002 |
| AM F69875 | *Thylacinus* sp. | Partial left dentary (fragmentary M_3_; M_4)_ | Big Sink Local Fauna; NSW | Late Pliocene (5-3) | Dawson et al., 1999 |
| UCMP 107737 | *Thylacinus* sp. | Partial P_2_ | Awe Local Fauna, New Guinea | Late Pliocene (4.0-3.0) | Plane, 1976 |
| SAM P29807 | *Thylacinus yorkellus* | Partial left dentary (C-P_3_; M_2-3_) | Curramulka Local Fauna, Corra-Lynn Cave, SA | Late Miocene - early Pliocene (5.3-3.6) | Pledge, 1992; Yates, 2015 |
| SAM P38799 | *Thylacinus yorkellus* | Partial right M_3_ | Curramulka Local Fauna, Corra-Lynn Cave, SA | Late Miocene - early Pliocene (5.3-3.6) | Yates, 2015 |
| NTM P98211 | *Tyarrpecinus rothi* | Partial left maxilla (P^2^; fragmentary M^1-4^) | Alcoota Local Fauna, Alcoota Station, NT | Late Miocene - early Pliocene (8.5-5.5) | Murray & Megirian, 2000 |
| QM F16851 | *Wabulacinus ridei* | Partial right maxilla (M^1-2^) | Camel Sputum Site, Godthelp Hill, Riversleigh, QLD | Early Miocene (18.5-17.0) | Muirhead, 1997 |
| QM F16852 | *Wabulacinus ridei* | Partial left dentary (M_3_) | Camel Sputum Site, Godthelp Hill, Riversleigh, QLD | Early Miocene (18.5-17.0) | Muirhead, 1997 |
| NTM P2815-10 | Thylacinidae *incertae sedis* | Partial right M^2^ | Pwerte Marnte Marnte Local Fauna; NT | Late Oligocene (>23.0) | Murray & Megirian, 2006a |
| QM F16809 | Thylacinidae *incertae sedis* (*Nimbacinus dicksoni* Muirhead & Archer, 1990) | Partial right M_2_ | D-Site, Riversleigh, QLD | Late Oligocene (>23.0) | Muirhead & Archer, 1990; Murray & Megirian 2000; Wroe & Musser 2001 |
| QM F16850 | Thylacinidae *incertae sedis* (*Thylacinus macknessi* Muirhead, 1992) | Right M^1^ | Dwornamor LF, Gag Site, Riversleigh, QLD | Middle Miocene (15.1-14.2) | Muirhead & Archer, 1990; Murray & Megirian 2000; Wroe & Musser 2001 |
| Dasyuromorphia *incertae sedis* | | | | | |
| NTM P907-3 | *Mutpuracinus archibaldi* | Partial left maxilla (P^2^-M^4^) | Blast Site, Bullock Creek, NT | Middle Miocene (17-12) | Murray & Megirian, 2000 |
| NTM P9612-5 | *Mutpuracinus archibaldi* | Partial left dentary (M_3-4_) | Top Site, Bullock Creek, NT | Middle Miocene (17-12) | Murray & Megirian, 2000 |
| NTM P9464-120 | *Mutpuracinus archibaldi* | Right premaxilla | Top Site, Bullock Creek, NT | Middle Miocene (17-12) | Murray & Megirian, 2000 |
| NTM P9464-119 | *Mutpuracinus archibaldi* | Partial right dentary | Top Site, Bullock Creek, NT | Middle Miocene (17-12) | Murray & Megirian, 2000 |
| NTM P87108-10 | *Mutpuracinus archibaldi* | Right M_4_ | Blast Site, Bullock Creek, NT | Middle Miocene (17-12) | Murray & Megirian, 2000 |
| NTM P91168-5 | *Mutpuracinus archibaldi* | Partial cranium | Jaw Junction Site, Riversleigh, QLD | Mid-late Miocene (13.7-7.3) | Murray & Megirian, 2006b |
