# Supplementary material for: The pre-Pleistocene fossil thylacinids (Dasyuromorphia: Thylacinidae) and the evolutionary context of the modern thylacine
Source: PeerJ. 2019 Sep 2;7:e7457. doi: 10.7717/peerj.7457 (PMC6727838; doi:10.7717/peerj.7457)
Supplement: Supplemental Information 2 — Mass estimated using Dasyuromorphia-only regressions from Myers (2001). Estimate includes smearing factor. SEE%, percent standard error of the estimate; PE%, percent prediction error; SE%, smearing estimate. All mass estimates from current study except 1Wroe (2001) and 2Yates (2014). [file peerj-07-7457-s002.docx]

| Taxon | Specimen | Variable (*x*) | Equation | SEE% | PE% | SE% | Metric (mm) | Mass (kg) |
| --- | --- | --- | --- | --- | --- | --- | --- | --- |
| *Badjcinus turnbulli*^1^ | QM F30407 | LMRL | log *y* = -1.075 + 3.209(log *x*) | 18 | 13 | 3.0 | 24.3 | 2.4 |
| *Badjcinus turnbulli* | QM F30408 | 2UMA | log *y* = 0.426 + 1.890(log *x*) | 31 | 21 | 2.9 | 41 | 3.1 |
| *Badjcinus turnbulli* | QM F30410 | 4LML | log *y* = 0.771 + 3.116(log *x*) | 37 | 25 | 4.4 | 6.1 | 1.7 |
| *Badjcinus turnbulli* | QM F30411 | 3LML | log *y* = 0.567 + 3.400(log *x*) | 33 | 22 | 3.5 | 6.8 | 2.6 |
| *Maximucinus muirheadae*^1^ | QM F30331 | 2UMW | log *y* = 0.379 + 4.038(log *x*) | 31 | 21 | 3.0 | 9.1 | 18.4 |
| *Muribacinus gadiyuli*^1^ | QM F30385 | LMRL | log *y* = -1.075 + 3.209(log *x*) | 18 | 13 | 3.0 | 21.2 | 1.6 |
| *Muribacinus gadiyuli* | QM F30386 | 2UMA | log *y* = 0.426 + 1.890(log *x*) | 31 | 21 | 2.9 | 30.2 | 1.7 |
| *Ngamalacinus timmulvaneyi*^1^ | QM F16853 | LMRL | log *y* = -1.075 + 3.209(log *x*) | 18 | 13 | 3.0 | 31.8 | 5.7 |
| *Ngamalacinus timmulvaneyi* | QM F16855 | 2UMA | log *y* = 0.426 + 1.890(log *x*) | 31 | 21 | 2.9 | 70.1 | 8.4 |
| *Ngamalacinus timmulvaneyi* | QM F30300 | 2UMA | log *y* = 0.426 + 1.890(log *x*) | 31 | 21 | 2.9 | 63.4 | 7.0 |
| *Nimbacinus dicksoni* | NTM P85553-3 | 1LML | log *y* = 0.723 + 3.688(log *x*) | 42 | 27 | 5.3 | 6.8 | 6.5 |
| *Nimbacinus dicksoni* | NTM P8695-92 | 4LML | log *y* = 0.771 + 3.116(log *x*) | 37 | 25 | 4.4 | 7.2 | 2.9 |
| *Nimbacinus dicksoni* | NTM P904-7 | 3LML | log *y* = 0.567 + 3.400(log *x*) | 33 | 22 | 3.5 | 8.0 | 4.5 |
| *Nimbacinus dicksoni* | NTM P9612-4 | LMORL | log *y* = -1.225 + 3.340(log *x*) | 20 | 16 | 1.6 | 32.2 | 6.6 |
| *Nimbacinus dicksoni* | QM F16802 | 1LML | log *y* = 0.723 + 3.688(log *x*) | 42 | 27 | 5.3 | 6.6 | 5.9 |
| *Nimbacinus dicksoni* | QM F16803 | 2UMA | log *y* = 0.426 + 1.890(log *x*) | 31 | 21 | 2.9 | 62.3 | 6.8 |
| *Nimbacinus dicksoni* | QM F16804 | 2UMA | log *y* = 0.426 + 1.890(log *x*) | 31 | 21 | 2.9 | 57.0 | 5.7 |
| *Nimbacinus dicksoni* | QM F16805 | 3UML | log *y* = 0.775 + 3.143(log *x*) | 35 | 23 | 4.0 | 7.8 | 3.9 |
| *Nimbacinus dicksoni* | QM F16806 | 3UML | log *y* = 0.775 + 3.143(log *x*) | 35 | 23 | 4.0 | 7.8 | 3.9 |
| *Nimbacinus dicksoni*^1^ | QM F36357 | LMRL | log *y* = -1.075 + 3.209(log *x*) | 18 | 13 | 3.0 | 30.5 | 5.0 |
| *Thylacinus macknessi*^1^ | QM F16848 | LMRL | log *y* = -1.075 + 3.209(log *x*) | 18 | 13 | 3.0 | 36.6 | 9.0 |
| *Thylacinus macknessi* | QM F19849 | 3LML | log *y* = 0.567 + 3.400(log *x*) | 33 | 22 | 3.5 | 9.0 | 6.7 |
| *Thylacinus megiriani* | NTM P9618 | 3UML | log *y* = 0.775 + 3.143(log *x*) | 35 | 23 | 4.0 | 17.4 | 49.1 |
| *Thylacinus potens* | CPC 6746 | UMORL | log *y* = -1.098 + 3.350(log *x*) | 19 | 14 | 1.2 | 55.1 | 55.0 |
| *Thylacinus potens* | NTM P4326 | UMRL | log *y* = -0.992 + 3.279(log *x*) | 18 | 14 | 1.2 | 51.0 | 41.0 |
| *Thylacinus potens* | NTM P4327 | LMRL | log *y* = -1.075 + 3.209(log *x*) | 18 | 13 | 3.0 | 63.3 | 52.3 |
| *Thylacinus potens* | UCMP 66206 | 3LML | log *y* = 0.567 + 3.400(log *x*) | 33 | 22 | 3.5 | 14.5 | 33.9 |
| *Thylacinus potens* | UCMP 66971 | 3UML | log *y* = 0.775 + 3.143(log *x*) | 35 | 23 | 4.0 | 14.6 | 28.3 |
| *Thylacinus* sp. | AM F69875 | 4LML | log *y* = 0.771 + 3.116(log *x*) | 37 | 25 | 4.4 | 15.7 | 32.8 |
| *Thylacinus* sp. | WPC 4506 | 3LML | log *y* = 0.567 + 3.400(log *x*) | 33 | 22 | 3.5 | 12.2 | 18.2 |
| *Thylacinus yorkellus*^2^ | SAM P29807 | 3LML | log *y* = 0.567 + 3.400(log *x*) | 33 | 22 | 3.5 | 12.0 | 17.8 |
| *Thylacinus yorkellus* | SAM P38799 | 3LML | log *y* = 0.567 + 3.400(log *x*) | 33 | 22 | 3.5 | 11.3 | 14.5 |
| *Tyarrpecinus rothi*^1^ | NTM P98211 | 1UML | log *y* = 0.290 + 3.945(log *x*) | 31 | 22 | 3.0 | 7.4 | 5.4 |
| *Wabulacinus ridei*^1^ | QM F16851 | 2UMW | log *y* = 0.379 + 4.038(log *x*) | 31 | 21 | 3.0 | 6.7 | 5.3 |
| *Wabulacinus ridei* | QM F16852 | 3LML | log *y* = 0.567 + 3.400(log *x*) | 33 | 22 | 3.5 | 9.4 | 7.8 |
