# Supplementary material for: The pre-Pleistocene fossil thylacinids (Dasyuromorphia: Thylacinidae) and the evolutionary context of the modern thylacine
Source: PeerJ. 2019 Sep 2;7:e7457. doi: 10.7717/peerj.7457 (PMC6727838; doi:10.7717/peerj.7457)
Supplement: Supplemental Information 3 [file peerj-07-7457-s003.docx]

Table S3. Genbank accession numbers for the 12S RNA molecular data.

| Taxon | Genbank Accession Number |
| --- | --- |
| *Antechinus flavipes* | KJ868098 |
| *Dasycercus cristicauda* | KJ868107 |
| *Dasyurus hallucatus* | NC_007630 |
| *Dasyurus maculatus* | KJ780029 |
| *Perameles nasuta* | KJ868137 |
| *Phascogale tapoatafa* | NC_006523 |
| *Thylacinus cynocephalus* | NC_011944 |
