# Supplementary material for: The pre-Pleistocene fossil thylacinids (Dasyuromorphia: Thylacinidae) and the evolutionary context of the modern thylacine
Source: PeerJ. 2019 Sep 2;7:e7457. doi: 10.7717/peerj.7457 (PMC6727838; doi:10.7717/peerj.7457)
Supplement: Supplemental Information 7 [file peerj-07-7457-s007.docx]

Dataset and age ranges for dating analyses:

FAD LAD
Djarthia_murgonensis 54.7 54.6
Perameles_nasuta 0 0
Antechinus_flavipes 0 0
Badjcinus_turnbulli 27.8 23.0
Barinya_wangala 23.03 11.62
Dasycercus_cristicauda 0 0
Dasyuroides_achilpatna 2.9 2.5
Dasyurus_dunmalli 4.2 3.6
Dasyurus_hallucatus 0 0
Dasyurus_maculatus 0 0
Muribacinus_gadiyuli 15.1 12.9
Mutpuracinus_archibaldi 17.0 12.0
Ngamalacinus_timmulvaneyi 18.5 16.2
Nimbacinus_dicksoni 17.0 12.0
Phascogale_tapoatafa 0 0
Sminthopsis_floravillensis 2.58 0.1
Sminthopsis_murina 0 0
Thylacinus_cynocephalus 0 0
Thylacinus_macknessi 18.5 16.2
Thylacinus_megiriani 7.5 4.5
Thylacinus_potens 8.5 5.5
Thylacinus_yorkellus 5.3 3.6
Tyarrpecinus_rothi 8.5 5.5
Wabulacinus_ridei 18.5 17.0

Newick tree for strap analysis in Fig. S1a:

(Djarthia_murgonensis,(Perameles_nasuta,((Mutpuracinus_archibaldi,Barinya_wangala,(Sminthopsis_murina,Dasyuroides_achilpatna,(Antechinus_flavipes,Phascogale_tapoatafa),(Sminthopsis_floravillensis,Dasyurus_dunmalli,(Dasycercus_cristicauda,(Dasyurus_hallucatus,Dasyurus_maculatus))))),(Badjcinus_turnbulli,((Nimbacinus_dicksoni,(Muribacinus_gadiyuli,Ngamalacinus_timmulvaneyi)),(Tyarrpecinus_rothi,(Wabulacinus_ridei,(Thylacinus_macknessi,(Thylacinus_potens,(Thylacinus_yorkellus,Thylacinus_cynocephalus,Thylacinus_megiriani))))))))));

Newick tree for strap analysis in Fig. S1b:

(Djarthia_murgonensis,(Perameles_nasuta,((((((Antechinus_flavipes,Phascogale_tapoatafa),(((Dasycercus_cristicauda,Dasyurus_dunmalli),(Dasyurus_hallucatus,Dasyurus_maculatus)),Sminthopsis_floravillensis)),(Dasyuroides_achilpatna,Sminthopsis_murina)),Mutpuracinus_archibaldi),Barinya_wangala),(Badjcinus_turnbulli,(((Muribacinus_gadiyuli,Nimbacinus_dicksoni),Ngamalacinus_timmulvaneyi),((((Thylacinus_cynocephalus,Thylacinus_yorkellus),Thylacinus_megiriani),Thylacinus_macknessi),(Thylacinus_potens,(Tyarrpecinus_rothi,Wabulacinus_ridei))))))));

Age ranges and references for fossil taxa.

| Taxon | Locality | Age Range (Ma) | Reference(s) |
| --- | --- | --- | --- |
| *Djarthia murgonensis* | Tingamarra Local Fauna, Etadunna Formation, South Australia | 54.7-54.6 | Godthelp et al. 1992 |
| *Badjcinus turnbulli* | see text for details | 27.8-23.0 | see text for details |
| *Barinya wangala* | Riversleigh Faunal Zones B & C, Queensland, Australia | 23.03-11.62 | Arena et al. 2015 |
| *Dasyuroides achilpatna* | Fisherman’s Cliff Local Fauna, Moorna Formation, New South Wales, Australia | 2.9-2.5 | Whitelaw, 1991 |
| *Dasyurus dunmalli* | Chinchilla Local Fauna, Queensland, Australia | 4.2-3.6 | Tedford et al., 1992 |
| *Muribacinus gadiyuli* | see text for details | 15.1-12.9 | see text for details |
| *Mutpuracinus archibaldi* | see text for details | 17.0-12.0 | see text for details |
| *Ngamalacinus timmulvaneyi* | see text for details | 18.5-16.2 | see text for details |
| *Nimbacinus dicksoni* | see text for details | 17.0-12.0 | see text for details |
| *Sminthopsis floravillensis* | Site 5C, Floraville Crossing, Queensland, Australia | 2.58-0.1 | Price, 2013 |
| *Thylacinus macknessi* | see text for details | 18.5-16.2 | see text for details |
| *Thylacinus megiriani* | see text for details | 7.5-4.5 | see text for details |
| *Thylacinus potens* | see text for details | 8.5-5.5 | see text for details |
| *Thylacinus yorkellus* | see text for details | 5.3-3.6 | see text for details |
| *Tyarrpecinus rothi* | see text for details | 8.5-5.5 | see text for details |
| *Wabulacinus ridei* | see text for details | 18.5-17.0 | see text for details |

References

Arena DA, Travouillon KJ, Beck R, Black KH, Gillespie AK, Myers TJ, Archer M, and Hand SJ. 2015. Mammalian lineages and the biostratigraphy and biochronology of Cenozoic faunas from the Riversleigh World Heritage Area, Australia. *Lethaia* 49:43-60. 10.1111/let.12131

Godthelp H, Archer M, Cifelli R, Hand SJ, and Gilkeson CF. 1992. Earliest known Australian Tertiary mammal fauna. *Nature* 356:514-516. 10.1038/356514a0

Price GJ. 2013. Quaternary. In: Jell PA, ed. *Geology of Queensland*. Brisbane, Queensland: State of Queensland, 653-686.

Tedford RH, Wells RT, and Barghoorn SF. 1992. Tirari formation and contained faunas, Pliocene of the Lake Eyre basin, South Australia. *The Beagle: Records of the Museums and Art Galleries of the Northern Territory* 9:173-193.

Whitelaw MJ. 1991. Magnetic polarity stratigraphy of the Fisherman's Cliff and Bone Gulch vertebrate fossil faunas from the Murray Basin, New South Wales, Australia. *Earth and Planetary Science Letters* 104:417-423. 10.1016/0012-821X(91)90219-8
