# Supplementary material for: The pre-Pleistocene fossil thylacinids (Dasyuromorphia: Thylacinidae) and the evolutionary context of the modern thylacine
Source: PeerJ. 2019 Sep 2;7:e7457. doi: 10.7717/peerj.7457 (PMC6727838; doi:10.7717/peerj.7457)
Supplement: Supplemental Information 8 — Modified from matrices presented in Yates (2014) and Kealy & Beck (2017). [file peerj-07-7457-s008.docx]

CHARACTER STATES
Modified from matrices presented in Yates (2014) and Kealy & Beck (2017).

1. Upper incisor number. Wroe & Musser (2001) ch. #1.
 (0) 5
 (1) 4

2. Morphology of C1. Wroe & Musser (2001) ch. #4.
 (0) caniform
 (1) premolariform

3. Height of P3 (ordered). Wroe & Musser (2001) ch. #5.
 (0) higher crowned than P2
 (1) subequal in height
 (2) lower crowned than P2
 (3) absent

4. Shape of P3 in occlusal view. Wroe & Musser (2001) ch. #6.
 (0) laterally compressed – markedly longer than wide
 (1) bulbous and ovate – not markedly longer than wide

5. Position of P2. Yates (2014) ch. #6.
 (0) P2 closer to P3 than P1
 (1) P2 equidistant between P1 and P3

6. Mesiodistal length of P2. Yates (2014) ch. #7.
 (0) P2 shorter than M1
 (1) P2 longer than M1

7. Mesiodistal length of P3. Yates (2014) ch. #8.
 (0) P3 shorter than M1
 (1) P3 longer than M1

8. Distolingual cuspule on P3. Wroe & Musser (2001) ch. #7.
 (0) absent
 (1) present

9. Relative size of paracone and metacone on M2-3. Modified from Wroe & Musser (2001) ch. #8.
 (0) metacone larger than paracone
 (1) Paracone and metacone are subequal in size

10. Metacone on M4 (ordered). Wroe & Musser (2001) ch. #9.
 (0) present and distinct from metastylar corner of tooth
 (1) present but not distinct from metastylar corner of tooth
 (2) absent

11. Shape and orientation of the centrocrista. Modified from Wroe & Musser (2001) ch. #10 & 11.
 (0) centrocrista strongly v-shaped
 (1) centrocrista weakly v-shaped
 (2) centrocrista incomplete, breaching the ectoloph
 (3) centrocrista straight, with apex well above the talon basin

12. Preparacrista on M1. Modified from Wroe & Musser (2001) ch. #12.
 (0) present
 (1) absent

13. Orientation of preparacrista on M1 (ordered). Modified from Wroe & Musser (2001) ch. #12.
 (0) M1 preparacrista forms a near perpendicular angle with respect to long axis of tooth
 (1) M1 preparacrista oriented mesiobuccally relative to long axis of tooth
 (2) M1 preparacrista runs distobuccally relative to long axis of tooth

14. Development of precingulum on M1 (ordered). Muirhead & Wroe (1998) ch. #12.
 (0) precingulum complete and extending from the mesiobuccal corner to a point anterior to the base of the protocone
 (1) precingulum incomplete, extending from mesiobuccal corner to a point anterior to the base of the paracone
 (2) precingulum absent

15. Presence/absence of precingulum on M3. Yates (2014) ch. #11.
 (0) present
 (1) absent

16. Development of ectoflexus on M2-3. Yates (2014) ch. # 14.
 (0) well-developed
 (1) extremely reduced or absent

17. Relative lengths of M3 and M4 preparacristae. Wroe & Musser (2001) ch. #13.
 (0) M4 preparacristae longer than that of M3
 (1) M4 preparacristae shorter than or equal to that of M3

18. Size of stylar cusp B on M3 (ordered). Wroe & Musser (2001) ch. #17.
 (0) large
 (1) small
 (2) absent

19. Relative size of stylar cusp B and stylar cusp D on M2 (ordered). Modified from Wroe & Musser (2001) ch. #18 & 19.
 (0) stylar cusp D present but smaller or subequal to stylar cusp B
 (1) stylar cusp present and much larger than stylar cusp B
 (2) stylar cusp D absent

20. Relationship of stylar cusp D to metacone. Wroe & Musser (2001) ch. #20.
 (0) not appressed
 (1) appressed

21. Elongation of the postmetacrista in upper molars (ordered). Modified from Muirhead & Wroe (1998) ch. #14.
 (0) not elongate, with the metastylar wing occupying 40-48% of the tooth length
 (1) mildly elongate, with the metastylar wing occupying 48-52% of the tooth length
 (2) strongly elongate, with the metastylar wing occupying >52% of the tooth length

22. Size of M3 relative to M2. Yates (2014) ch. #21.
 (0) M3 and M2 are subequal
 (1) M3 distinctly larger than M2

23. Shape of M3. Yates (2014) ch. #22.
 (0) M3 as wide as, or wider than it is long
 (1) M3 longer than wide

24. Size of stylar cusp B on M1-2. Modified from Muirhead & Wroe (1998) ch. #11.
 (0) well-developed
 (1) highly reduced or absent

25. Development of stylar cusp D on M2 (ordered). Yates (2014) ch. #26.
 (0) present and large
 (1) reduced to a slight bulge or bump
 (2) absent

26. Presence of absence of stylar cusp D on M3. Modified from Wroe & Musser (2001) ch. #18.
 (0) present
 (1) absent

27. Presence of absence of stylar crest on M3. Modified from Muirhead (1997) ch. #7.
 (0) present
 (1) absent

28. “Central cusp” (ordered). Wroe & Musser (2001) ch. #22.
 (0) present
 (1) absent

29. Lower incisor number. Wroe & Musser (2001) ch. #26.
 (0) 4
 (1) 3

30. Morphology of i3. Wroe & Musser (2001) ch. #27.
 (0) not bilobed
 (1) bilobed

31. Morphology of i2. Modified from Wroe & Musser (2001) ch. #29.
 (0) not staggered
 (1) staggered

32. Diastema between p1-2. Muirhead & Wroe (1998) ch. #22.
 (0) present
 (1) absent

33. Diastema between p2-3. Muirhead & Wroe (1998) ch. #23.
 (0) absent
 (1) present

34. Height of p3 relative to p2 (ordered). Wroe & Musser (2001) ch. #43.
 (0) p3 higher crowned than p2
 (1) subequal in height
 (2) smaller than p2
 (3) absent

35. Mesiodistal length of p3 relative to p2. Modified from Muirhead & Wroe (1998) ch. #30.
 (0) p3 shorter
 (1) p3 longer

36. Diastema between p3-m1. Yates (2014) ch. #32.
 (0) absent
 (1) present

37. Presence of hypoconulid notch. Wroe & Musser (2001) ch. #30.
 (0) present
 (1) absent

38. Entoconid size. Wroe & Musser (20010 ch. #41.
 (0) large
 (1) reduced or absent

39. Development of metaconid in m1 (ordered). Modified from Muirhead & Wroe (1998) ch. #18.
 (0) well-developed, distinct cusp
 (1) reduced to a small cuspule on the side of the protoconid
 (2) absent

40. Size of metaconid on m1 relative to that of m2-4. Wroe & Musser (2001) ch. #32.
 (0) metaconid of m1 not reduced relative to that of m2-4
 (1) metaconid of m1 reduced relative to that of m2-4

41. Size of metaconid in m2-4 (ordered). Wroe & Musser (20010) ch. #33.
 (0) large
 (1) reduced
 (2) absent

42. Size of paraconid on m1 (ordered). Modified from Wroe & Musser (2001) ch. #34.
 (0) large
 (1) reduced, but still identifiable as a distinct cusp
 (2) absent

43. Distal cingulid in m1-3 (ordered). Wroe & Musser (2001) ch. #36.
 (0) present
 (1) absent

44. Presence of a carnassial notch in the cristid obliqua. Muirhead & Wroe (1998) ch. #26.
 (0) absent
 (1) present

45. Anterior point of termination of the cristid obliqua in m3 relative to carnassial notch formed by postprotocristid and metacristid (ordered). Modified from Wroe & Musser (2001) ch. #40.
 (0) buccal to carnassial notch
 (1) beneath carnassial notch
 (2) lingual to carnassial notch

46. Relative size of m4 to that of m3. Modified from Wroe & Musser (2001) ch. #42.
 (0) m4 smaller than m3
 (1) m4 subequal or larger than m3

47. Number of distinct cusps on m4 talonid (ordered). Wroe & Musser (2001) ch. #44.
 (0) 3
 (1) 2
 (2) 1

48. Relationship of jugal to infraorbital foramen (ordered). Modified from Muirhead & Wroe (1998) ch. #3.
 (0) jugal widely separated from the margin of the infraorbital foramen
 (1) maxillojugal suture passes very close to the margin of the infraorbital foramen
 (2) jugal contributes to the posterior margin of the infraorbital foramen

49. Position of the infraorbital foramen. Murray (1997) ch. #12.
 (0) dorsal to M1
 (1) dorsal to M2

50. Presence of orbital crest. Wroe & Musser (2001) ch. #45.
 (0) absent
 (1) present

51. Presence of maxillopalatine fenestrae. Modified from Voss & Jansa (2003) ch. #38.
 (0) absent
 (1) present

52. Presence of palatine fenestrae. Voss & Jansa (2003) ch. #39.
 (0) absent
 (1) present

53. Presence of anterior palatal fenestrae. From Kealy & Beck (2017), following Voss & Jansa (2003).
 (0) absent
 (1) present

54. Presence of complete posterolateral palatine foramen. Wroe & Musser (2001) ch. #48.
 (0) present
 (1) absent

55. Presence of accessory posterolateral palatine foramen. Wroe & Musser (2001) ch. #49.
 (0) present
 (1) absent

56. Contribution of alisphenoid and periotic to primary foramen ovale. Wroe & Musser (2001) ch. #50.
 (0) delimited y alisphenoid anteriorly and periotic part of the petrosal posteriorly
 (1) delimited by alisphenoid only

57. Presence of secondary foramen ovale formed by anteriorly directed strut of alisphenoid tympanic process (ordered). Wroe & Musser (2001) ch. #51.
 (0) absent
 (1) present but incomplete
 (2) present and complete

58. Presence of secondary foramen ovale formed by mesial fold in alisphenoid tympanic process (ordered). Wroe & Musser (2001) ch. #52.
 (0) absent
 (1) present but incomplete
 (2) present and complete

59. Presence of contribution to secondary foramen ovale by posteriorly directed strut in alisphenoid (ordered). Wroe & Musser (2001) ch. #55.
 (0) absent
 (1) present but incomplete
 (2) present and complete

60. Ventral facial nerve canal. Modified from Wroe & Musser (2001) ch. #54.
 (0) absent
 (1) present

61. Squamosal epitympanic sinus. Modified from Wroe & Musser (2001) ch. #55.
 (0) absent
 (1) present

62. Bones contributing to the hypotympanic sinus roof. Modified from Wroe & Musser (2001) ch. #56.
 (0) alisphenoid and petrosal
 (1) alisphenoid only
 (2) alisphenoid, petrosal, and squamosal

63. Size of alisphenoid tympanic process (ordered). Wroe & Musser (2001) ch. #57.
 (0) absent
 (1) poorly developed
 (2) well developed

64. Length of the internal jugular canal. Wroe & Musser (2001) ch. #60.
 (0) does not extend anteriorly to the basisphenoid
 (1) extends to the basisphenoid

65. Presence of a well developed posteroventral lip formed by a mesially directed process in the pars petrosal, enclosing the internal jugular ventrally. Wroe & Musser (2001) ch. #61.
 (0) absent
 (1) present

66. Presence of transverse canal. Wroe & Musser (2001) ch. #61.
 (0) absent
 (1) present

67. Frontal-squamosal or alisphenoid-parietal contact on the lateral wall of the braincase. Modified from Wroe & Musser (2001) ch. #64.
 (0) alisphenoid-parietal contact
 (1) frontal-squamosal contact

68. Morphology of the rostral tympanic process of the petrosal (ordered). Modified from Wroe & Musser (2001) ch. #65.
 (0) absent
 (1) present but small, not enclosing a sinus
 (2) present and large, enclosing a distinct sinus

69. Paroccipital process. Modified from Wroe & Musser (2001) ch. #66.
 (0) absent
 (1) present

70. Morphology of foramen for the greater petrosal nerve. Modified from Wroe & Musser (2001) ch. #69.
 (0) distinct from primary foramen ovale
 (1) confluent with primary foramen ovale

71. Tubal foramen. Modified from Wroe & Musser (2001) ch. #70.
 (0) absent
 (1) present

72. Morphology of the tubal foramen. Modified from Wroe & Musser (2001) ch. #70.
 (0) slit-like and incomplete anteroventrally
 (1) ovoid, with sulcus in pars squamosal
 (2) sulcus in alisphenoid tympanic process

73. Shape of nasals. Wroe & Musser (2001) ch. #71.
 (0) nasals posteriorly expanded
 (1) not posteriorly expanded

74. Maxilla-nasal contact. Wroe & Musser (2001) ch. #72.
 (0) maxilla-nasal contact longer than premaxilla-nasal contact
 (1) premaxilla-nasal contact longer than maxilla-nasal contact

75. Posterior extension of nasals. Wroe & Musser (2001) ch. #73.
 (0) nasals extend posteriorly beyond the anterior rim of the orbit
 (1) nasals do not extend posteriorly beyond the orbit

76. Frontal-maxillary contact. Wroe & Musser (2001) ch. #74.
 (0) present
 (1) absent

77. Morphology of jugal. Wroe & Musser (2001) ch. #75.
 (0) jugal not Y-shaped
 (1) jugal Y-shaped

78. Antorbital fossa. Modified from Wroe & Musser (2001) ch. #76.
 (0) absent
 (1) present

79. Presence of prootic canal. Wroe & Musser (2001) ch. #77.
 (0) present
 (1) absent

80. Body size (ordered). Yates (2014) ch. 42 following Murray (1997).
 (0) dental metrics consistent with body mass <15 kg
 (1) dental metrics consistent with body mass 15-35 kg
 (2) dental metrics consistent with body mass >35 kg

Kealy S, and Beck R. 2017. Total evidence phylogeny and evolutionary timescale for Australian faunivorous marsupials (Dasyuromorphia). *BMC Evolutionary Biology* 17:1-23.

Muirhead J. 1997. Two new early Miocene thylacines from Riversleigh, northwestern Queensland. *Memoirs of the Queensland Museum* 41:367-378.

Muirhead J, and Wroe S. 1998. A new genus and species, *Badjcinus turnbulli* (Thylacinidae: Marsupialia), from the late Oligocene of Riversleigh, northern Australia, and an investigation of thylacinid phylogeny. *Journal of Vertebrate Paleontology* 18:612-626.

Murray PF. 1997. *Thylacinus megiriani*, a new species of thylacine (Marsupialia: Thylacinidae) from the Ongeva local fauna of central Australia. *Records of the South Australian Museum* 30:43-61.

Voss RS, and Jansa SA. 2003. Phylogenetic studies on didelphid marsupials II. Nonmolecular data and new IRBP sequences: separate and combined analyses of didelphine relationships with denser taxon sampling. *Bulletin of the American Museum of Natural History*:1-82. 10.1206/0003-0090(2003)276<0001:PSODMI>2.0.CO;2

Wroe S, and Musser A. 2001. The skull of *Nimbacinus dicksoni* (Thylacinidae: Marsupialia). *Australian journal of Zoology* 49:487-514.

Yates AM. 2014. New craniodental remains of *Thylacinus potens* (Dasyuromorphia: Thylacinidae), a carnivorous marsupial from the late Miocene Alcoota Local Fauna of central Australia. *PeerJ* 2:e547.
