# Supplementary figures and images for: The pre-Pleistocene fossil thylacinids (Dasyuromorphia: Thylacinidae) and the evolutionary context of the modern thylacine
Source: PeerJ. 2019 Sep 2;7:e7457. doi: 10.7717/peerj.7457 (PMC6727838; doi:10.7717/peerj.7457)

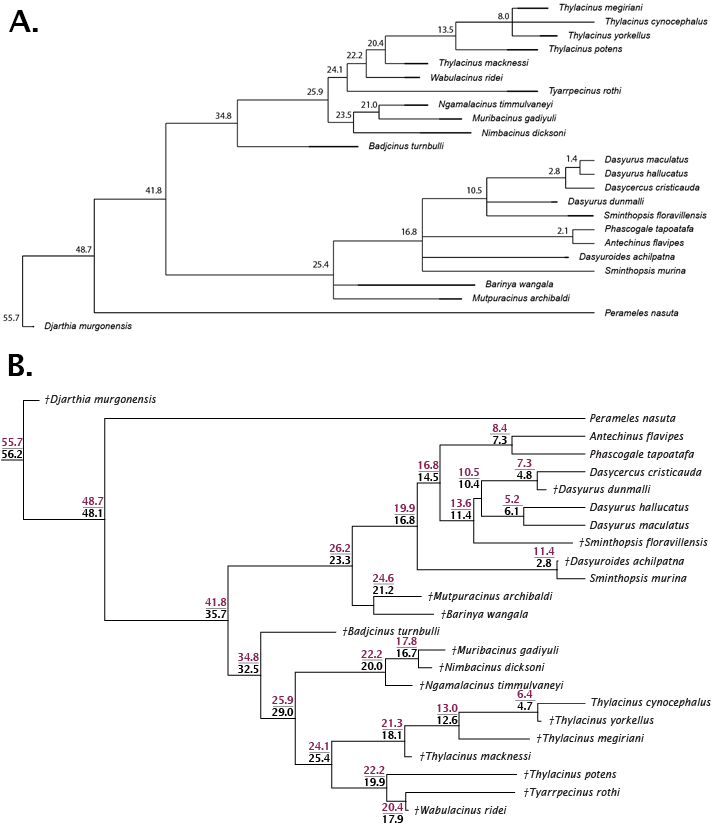

Supplement: Supplemental Information 11 — (A) Age estimate for the parsimony analysis. Tree generated by implicit enumeration in TNT and fed to strap along with FAD/LAD of taxa. Numbers are years (Ma). Node age estimate comparison for strap and Bayesian FBD analyses. Tree generated by Bayesian tip-dated FBD analysis and fed to strap along with FAD/LAD of taxa. Numbers at nodes are node ages (Ma), top (purple) is strap, bottom (black) is Bayesian. Data in Dataset S4, R code in File S2. [file peerj-07-7457-s011.png]
